# Health data sharing attitudes towards primary and secondary use of data: a systematic review

**DOI:** 10.1016/j.eclinm.2024.102551

**Published:** 2024-03-18

**Authors:** Fidelia Cascini, Ana Pantovic, Yazan A. Al-Ajlouni, Valeria Puleo, Lucia De Maio, Walter Ricciardi

**Affiliations:** a Department of Life Sciences and Public Health, Section of Hygiene and Public Health, Università Cattolica del Sacro Cuore, L. go Francesco Vito 1, Rome, 00168, Italy; b Faculty of Biology, University of Belgrade, Belgrade, Serbia; c New York Medical College School of Medicine, Valhalla, NY, USA; d Directorate General for the Digitisation of the Health Information System and Statistics, Ministry of Health, Italy

**Keywords:** Data sharing, Personal health data, Secondary use, Genomic data, Biobank, Person-generated data

## Abstract

**Background:**

To receive the best care, people share their health data (HD) with their health practitioners (known as sharing HD for primary purposes). However, during the past two decades, sharing for other (i.e., secondary) purposes has become of great importance in numerous fields, including public health, personalized medicine, research, and development. We aimed to conduct the first comprehensive overview of all studies that investigated people's HD sharing attitudes—along with associated barriers/motivators and significant influencing factors—for all data types and across both primary and secondary uses.

**Methods:**

We searched PubMed, MEDLINE, PsycINFO, Web of Science, EMBASE, and CINAHL for relevant studies published in English between database inception and February 28, 2023, using a predefined set of keywords. Studies were included, regardless of their design, if they reported outcomes related to attitudes towards sharing HD. We extracted key data from the included studies, including the type of HD involved and findings related to: HD sharing attitudes (either in general or depending on type of data/user); barriers/motivators/benefits/concerns of the study participants; and sociodemographic and other variables that could impact HD sharing behaviour. The qualitative synthesis was conducted by dividing the studies according to the data type (resulting in five subgroups) as well as the purpose the data sharing was focused on (primary, secondary or both). The Newcastle–Ottawa Scale (NOS) was used to assess the quality of non-randomised studies. This work was registered with PROSPERO, CRD42023413822.

**Findings:**

Of 2109 studies identified through our search, 116 were included in the qualitative synthesis, yielding a total of 228,501 participants and various types of HD represented: person-generated HD (n = 17 studies and 10,771 participants), personal HD in general (n = 69 studies and 117,054 participants), Biobank data (n = 7 studies and 27,073 participants), genomic data (n = 13 studies and 54,716 participants), and miscellaneous data (n = 10 studies and 18,887 participants). The majority of studies had a moderate level of quality (83 [71.6%] of 116 studies), but varying levels of quality were observed across the included studies. Overall, studies suggest that sharing intentions for primary purposes were observed to be high regardless of data type, and it was higher than sharing intentions for secondary purposes. Sharing for secondary purposes yielded variable findings, where both the highest and the lowest intention rates were observed in the case of studies that explored sharing biobank data (98% and 10%, respectively). Several influencing factors on sharing intentions were identified, such as the type of data recipient, data, consent. Further, concerns related to data sharing that were found to be mutual for all data types included privacy, security, and data access/control, while the perceived benefits included those related to improvements in healthcare. Findings regarding attitudes towards sharing varied significantly across sociodemographic factors and depended on data type and type of use. In most cases, these findings were derived from single studies and therefore warrant confirmations from additional studies.

**Interpretation:**

Sharing health data is a complex issue that is influenced by various factors (the type of health data, the intended use, the data recipient, among others) and these insights could be used to overcome barriers, address people's concerns, and focus on spreading awareness about the data sharing process and benefits.

**Funding:**

None.

**Research in context**

**Evidence before this study**

Prior to conducting this study, we performed a search of all published reviews that explored health data sharing behaviour/intentions. The published reviews have so far attempted to investigate specific aspects related to attitudes or willingness to share health data by narrowing their selection criteria and focusing factors such as the type of use, the type of health data, type of study design and more. There was no systematic review that summarized all findings regarding willingness to share HD or information (regardless of the study designs), the corresponding motives and concerns, significant predictors, and to draw conclusions based on sharing for primary and secondary purposes. We aimed to fill this knowledge gap. On February 28, 2023, we used the following search terms (“health data” OR “patient data” OR “medical data” OR “health information” OR “person generated health data” OR “electronic health records” OR “mobile apps” OR “apps” OR “wearable technology” OR “wearable device” OR “big data”) AND (“sharing” OR “data sharing” OR “health data sharing” OR “health information exchange”) AND (“attitudes” OR “beliefs” OR “perceptions” OR “willingness”). This search yielded 2109 articles for screening, aiming to provide a thorough understanding of the current landscape in health data sharing intentions and behaviors.

**Added value of this study**

This systematic review included 116 studies, divided into five subgroups according to the data type that was investigated to be shared: person-generated HD (collected via sensors, devices, etc., n = 17), personal HD in general (n = 69), biobank data (n = 7), genomic data (n = 13), and the fifth subgroup reported miscellaneous data (n = 10). Overall, this systematic review provided the first comprehensive qualitative summary of findings related to sharing attitudes/behaviours (and associated predictors of this behaviour, and opinions of the study populations that were classified as being concerns/motivators that were related to sharing behaviour) depending on the data type and the type of use (primary or secondary).

**Implications of all the available evidence**

This thorough systematic review revealed that people are more open to share their data for the means of receiving care. Although a great number of studies concluded high support for some secondary uses (such as for non-commercial research), the attitude varies greatly depending on the type of data recipient. This review identified that people share the same concerns related to privacy and safety regardless of data type and that they would share their data as they perceive it will help improve healthcare and advance health knowledge. Overcoming barriers, addressing concerns, and spreading awareness about data sharing practices may lead to a more active data-sharing society.

## Introduction

To receive the best care, people readily share their health data (HD) with their health practitioners (which is known as sharing HD for primary purposes). However, during the past two decades, secondary health data (HD) use has experienced an expansion. Secondary purposes can include sharing for both clinical and public health research, publishing national statistics, education, developing algorithms, and others. Secondary HD use largely depends on people's willingness to share their data. This expansion started in 2002 when the World Medical Association's Declaration on Ethical Considerations regarding Health Databases recognized the importance of databases in health research, quality assurance and risk management,^1^ which generated demand for using HD for purposes outside of just with a general practitioner. The benefits of HD reuse were further acknowledged in 2006 by the Medical Research Council and Welcome Trust who emphasized the need for data sharing optimization for research purposes.^2^ So far, there have been several statements brought by eminent organizations such as the Welcome Trust and the Hewlett Foundation, that focused on increasing the availability of the data collected from the research they fund and promoting their use to facilitate public health research.^3^ Similar statements were published afterwards from the International Committee of Medical Journal Editors^4^ and the US Institute of Medicine^5^ where the focus was put on the “ethical obligation to share” and proposed that data sharing should be the expected norm.

However, a clarification about what HD refers to and the distinction between primary and secondary uses of HD should be made. According to the General Data Protection Regulation (GDPR), health data is defined as “all data pertaining to the health status of a data subject which reveal information relating to the past, current or future physical or mental health status of the data subject.”^6^ More specifically, HD includes a wide range of data such as those derived from medical examinations, lab tests, genetic data, those gathered via medical devices, mobile apps, etc. When it comes to the purpose for which HD can be shared, primary use of data is typically performed by the entities that produce or collect these data while providing real-time, direct care to the healthcare consumers, while secondary use of data refers to non-direct care use of health information, such as analysis, research, quality/safety measurement, public health, payment, provider certification or accreditation, education/teaching services, etc.^7^

Sharing health data can lead to improved data transparency, better research reproducibility,^8^ and increased cost-effectiveness as a result of minimizing the repetition of research work. As a result, science and clinical knowledge can advance and the process of finding effective and safe patient treatments can be accelerated.^8^ It is noteworthy to point to some of the technological advancements that are reported to be a result of data-sharing initiatives. For example, the sharing of primary data (such as those collected via Health Information Exchange (HIE) for secondary uses were seen to bring advances at the healthcare system level. The United Kingdom National Health Service (NHS) has placed a focus on continuing their digital transformation and their ability to share primary care data with secondary care clinicians. During the period between 2015 and 2019, the NHS evidenced a rapid evolution of interoperable technology that allowed data to easily be shared between primary and secondary care practitioners. According to one study, primary to secondary care data-sharing capabilities were associated with a reduction in the number of patients breaching an Accident & Emergency 4-h decision time threshold in addition to an improved experience for patients who reported improved quality of acute hospital care. This is one piece of evidence that confirms that improvement in data-sharing capacities can bring benefits in the quality and service in healthcare systems.^9^ Some of the latest technological advancements that are based on sharing health data and their reuse can be seen in the example of digital twins. Digital twins represent a digital model or replication of physical entities—for example, it can be a virtual replica of human organs, tissues, cells that is used for predicting corresponding future scenarios.^10^ Digital twins are seen to revolutionize healthcare systems by allowing integration of real-time data, advanced analytics, and virtual simulations that can lead to improved patient care, conducting predictive analytics, optimization of clinical operations, and performing training and simulation.^10^ Additionally, the synergy between artificial intelligence and big data are continuously leveraging novel algorithms that are used in disease prediction, diagnosis, or in predicting therapeutical outcomes, among other applications.^11^

Along with conveying the importance of the use of HD for secondary purposes is the importance of assuring public acceptance towards data sharing practices.^12^ In one part, this means that secondary health data use should be aligned with the interests of the public (donors of health data), and thus it needs to have public support. To enable efficient secondary use of health data, it is necessary that health data donors understand the aspects surrounding the secondary use of their data and that way enables their support. However, sharing for both primary and secondary purposes come with associated challenges. Empirical evidence shows that people express different intentions when sharing depending on whether they had to enclose their HD with healthcare professionals within the same department, hospital, or some institution outside their hospital chain.^13^^,^^14^ Sharing HD for secondary purposes can face even more challenges, since their reuse can be applied in a wide range of fields, and for example, studies found that people tend to change their attitude towards data use depending on whether they consider the secondary research ‘acceptable’ (e.g., health service) and ‘unacceptable’ (e.g., commercial) research.^15^, ^16^, ^17^ It also largely depends on the level of anonymity of HD, which can be either anonymous, de-identified, reversibly anonymous or identifiable information that are publicly available, and evidence suggests that people express higher support for sharing their HD with higher levels of privacy.^18^^,^^19^ Previous research that has dealt with the issue related to HD sharing, mainly focused on exploring the practitioners' views, while data addressing the public opinion on this topic was scarce.^20^ However, this has changed during the last decade, as an increasing number of studies that investigated public acceptability towards secondary use of HD have been published.^21^

Several systematic reviews have summarized the findings from both qualitative and quantitative studies that explored the issues related to the public attitude regarding HD sharing for primary and secondary uses. According to the published evidence, two systematic reviews concluded that the support for HD sharing was widespread, however, with certain conditions, and accompanied by concerns (e.g., privacy, transparency).^21^^,^^22^ Other authors collected evidence about significant sociodemographic predictors and concluded that male and older individuals expressed higher willingness to give their consent for secondary review of their medical data and consequent use for research.^15^ Another systematic review explored attitudes regarding sharing data for secondary research use by performing a thematic analysis and yielded four key themes: benefits, fears and harms of data sharing, data sharing processes (especially the role of consent), and the participants–research relationship.^23^ The published systematic reviews have so far been designed to explore specific aspects related to attitudes or willingness to share HD, and their selection criteria was narrow and focused on the type of use, the type of data or the type of study design. No systematic review has been aimed at summarizing all findings regarding willingness to share HD or information (regardless of the study designs), the corresponding motives and concerns and the significant predictors, then draw conclusions based on sharing for primary and secondary purposes.

Therefore, this systematic review represents the first comprehensive overview of all studies that explored attitudes or intentions of sharing HD, which provides a qualitative summary of these findings for all data types, depending on the type of use (primary or secondary). This systematic review adds value by summarizing the findings related to sharing intentions, and those related to underlying motivators, barriers, concerns expressed by the study participants, and significant predictors that were found to modulate and impact their sharing behaviour.

## Methods

### Search strategy and selection criteria

We conducted a comprehensive literature search following the PRISMA (Preferred Reporting Items for Systematic Reviews and Meta-Analyses) guidelines. We searched PubMed, MEDLINE, PsycINFO, Web of Science, EMBASE, and CINAHL for studies published in English between published in English between database inception and February 28, 2023, using a predefined set of keywords. This systematic review is reported following the PRISMA (Preferred Reporting Items for Systematic Reviews and Meta-Analyses) guidelines, and the PRISMA reporting checklist is included as Supplementary materials. The PROSPERO database contains the registered protocol for this systematic review under the registration number CRD42023413822. The necessary amendments and updates were made during the process of conducting this review and can be accessed via PROSPERO.

The researchers created a tailored set of search terms to align with the specific research questions of this study. Boolean operators were employed to optimize the search effectiveness within the chosen databases. Detailed information on the specific search terms used is presented in Supplementary Table S1. These keywords were utilised to retrieve relevant literature from the six databases.

The retrieved articles were imported into the Endnote reference manager software for duplicate removal, after which the final list of articles was imported in Rayyan software, and two investigators (Y.A.A and A.P) independently evaluated the titles and abstracts of the retrieved articles to determine their relevance. Conflicts were resolved either by reaching consensus or by contacting the third reviewer (F.C.). After excluding articles that were deemed irrelevant at this initial stage, full-text versions of the remaining articles were obtained and assessed for eligibility and screened in the same manner. Inclusion criteria for the study encompassed the following: 1) Studies reporting attitudes towards sharing HD, including motivators, barriers, and factors influencing data sharing; 2) Quantitative primary studies, including randomised controlled trials, quasi-RCTs, controlled before-and-after studies, cross-sectional studies, interrupted time series, cohort studies, case–control studies, and ecological studies; 3) Mixed-methods primary studies if the qualitative and quantitative sections were presented separately; 4) Qualitative studies reporting quantitative results related to willingness to share HD. The exclusion criteria were defined as follows: 1) Non-human studies; 2) Conference proceedings, studies lacking full-text accessibility, and non-primary studies (e.g., reviews, systematic reviews); 3) Studies that did not report any result of interest; 4) Studies that focused on sharing biospecimen, except when biospecimen were mentioned together with other HD of interest; 5) Studies that focused only on participating in research studies (with the exception of studies that also reported willingness to share HD, such as in biobank studies); 6) Studies that explored sharing behaviours of populations not of interest (such as only healthcare personnel); 7) Studies that explored sharing behaviour only with friends, family, peers, etc.; 8) Studies that were retracted; 9) Studies not written in the English language.

Finally, in instances where a single study or dataset was published in multiple publications, preference was given to the publication that presented relevant outcomes and additional pertinent information aligned with the research question. Additionally, priority was given to publications published in a more suitable format, such as original research articles, over research letters.

### Statistical analysis

After the screening stage, a structured table was created to facilitate data extraction. Two reviewers conducted the data extraction process using a predefined table that was reviewed by coauthors. The extracted data encompassed various aspects, such as author information, year of publication, study title, study design, description of the study sample, type of HD involved, duration and recruitment period, specific types of HD collected, purpose of data collection (e.g., primary or secondary use), study objective(s), and key findings—those related to HD sharing attitudes (either in general or depending on type of data/user), those related to barriers/motivators/benefits/concerns of the study participants, and those related to sociodemographic and other variables that were recognized to significantly impact HD sharing behaviour. When a publication reported extensive results both for primary and secondary purposes, this information was extracted and presented in two separate entries (one study was presented twice in two separate rows). In cases when the information for HD sharing attitudes for both primary and secondary uses was not extensive and was presented with the aim to compare them, then the study was presented as one entry, by summarizing both types of findings. Thus, the comparisons depending on the type of use were primarily carried out by comparing the findings from individual studies, except in the case of studies classified as those reporting findings regarding sharing for both types of uses, in which these comparisons were already carried out as that was the study aim. Furthermore, all other findings on the influence of sociodemographic and data-specific factors were presented by summarizing the findings from individual studies depending on the type of data use.

The results were categorized in the following way. First the studies were divided according to the type of data that was shared (Table 1: person/patient-generated health data; Table 2: personal health data/information; Table 3: biobank health data; Table 4: genomic and genetic health data; Table 5: miscellaneous types of data). Further, they were divided (and reported in the Table 1, Table 2, Table 3, Table 4, Table 5) depending on the purpose the sharing was focused on (primary, secondary, both primary and secondary). The results concerning the rates of willingness to share are presented in a descending way, starting from those that reported the highest rates. After those studies, we presented the studies that reported variability in sharing intentions depending on data type, data user, and similar factors. Finally, at the end we also enlisted the studies that did not report rates of sharing intentions (but only the findings concerning significant influencing variables and concerns/benefits presented in Tables 6 and 7), and presented their summary characteristics, which is in accordance with the PRISMA 2020 checklist requirements. Other results regarding identified concerns and benefits are presented in Table 6, and those concerning significant sociodemographic and data-specific variables are presented in Table 7. and data specific variables are presented in Table 6 and those concerning identified concerns and benefits are presented in Table 7. Here we also need to mention the semantics issue we observed in studies from the second subgroup: personal health information and personal HD were used interchangeably, and the term personal medical information was used in the same context as personal health information. Therefore, we categorized these data as personal HD and information (PHID) in general. We aimed to explore potential differences between electronic vs non-electronic data, thus we made a distinction for all data types whether the data was electronic (when it was specifically mentioned that data sharing was performed electronically, or the data is digital); when no such details were provided, we did not make any specifications. Further, in case of the 2nd subgroup (which includes studies that reported sharing different types of PHID), since the HIE includes technologies that support capturing and sharing of all electronic healthcare information (not only Electronic Health Records (EHRs),^18^ we also made a distinction between PHID for HIE, and PHID stored in either EHRs, Electronic Medical Records (EMRs), Personal Health Records (PHRs) or Personally Controlled Health Records (PCHRs). The distinction between EHRs, EMRs, PHRs was also necessary since these records differ between each other in some respects.^24^^,^^25^ When commenting on the findings of the summarized studies, general willingness to share some type of data was presented as such, but when this result was presented depending on the user, type of data, consent or similar, it was used as a factor that impacts the willingness to share. Finally, we should also highlight what different categories of HD use purposes mean:

1) Primary use: those uses directed to patient care, referring to sharing HD with healthcare personnel.
2) Secondary use: uses that are directed to purposes other than patient care, such as research, education, public health, often requiring the sharing of HD with universities, pharmaceutical or insurance companies, and more.

**Table 1:** Summary characteristics of studies reporting findings related to sharing behavior of person/patient-generated health data.

Reference/Title	Type of HD/Purpose for data collection (primary vs secondary)	Study population	Duration/period of the recruitment for study sample/Geographical location	Study design	Aim	Attitudes/behavior/willingness towards sharing HD
**Studies reporting sharing behavior/attitudes/intentions for primary purposes**						
Runkle et al., 2019/Use of wearable sensors for pregnancy health and environmental monitoring: Descriptive findings from the perspective of patients and providers	Health data from wearable sensors (electronic data)/Primary	28 family medicine or obstetrics and gynecology providers at a rural health clinic and 103 pregnant women attending the clinic for prenatal care	January–April 2017/Western North Carolina, USA	Survey-based study	The purpose of this study was to examine the perceptions of pregnant women and their providers at a rural health clinic on the use of wearable technology to monitor health and environmental exposures during pregnancy.	A large majority of pregnant women in this sample (n = 95, 93%) would feel comfortable sharing information from personal monitoring devices with their doctor.
Luo et al., 2020/Interrelationships between patients' data tracking practices, data sharing practices, and health literacy: Onsite survey study	Patient-generated data (electronic and non-electronic data)/Primary	109 patients. 86 (78.9%) were female, and their ages ranged from 18 to 37 years (mean 21.0, SD 3.82).	NR/USA	Survey-based study	This study aims to systematically examine the interrelationships between patient-generated data tracking practices, data sharing practices, and health literacy among individual patients.	A total of 85.3% of participants, including 11 non-trackers, reported that they had shared their patient-generated data with their clinicians during the visit.
Rising et al., 2021/Willingness to share data from wearable health and activity trackers: Analysis of the 2019 health information national trends survey data	Health data from wearable health and activity trackers (electronic data)/Primary	1300 wearable health and activity tracker users; there were 55.03% of women, the majority were under 50 years of age (64.1%).	January and April 2019/USA	Survey-based study	This study aims to identify sociodemographic, health, and digital health behavior correlates of US adults' willingness to share wearable data with health care providers and family or friends.	Most US adults reported willingness to share wearable data with providers (81.86%) and with family or friends (69.51%).
Brown et al., 2022/Collecting and sharing self-generated health and lifestyle data: Understanding barriers for people living with long-term health conditions - a survey study	Self-generated health and lifestyle data (electronic and non-electronic data)/Primary	250 UK adults who reported living with a range of long-term health conditions. 66.4% were women, ages ranged from 18 to 77 years (M = 39.20, SD = 14.78).	NR/UK	Survey-based study	To assess self-reported behaviors, experiences, attitudes, and motivations relevant to sharing self-generated health and lifestyle data.	Of those who reported sharing their self-generated health and lifestyle data with others (n = 202), 74% reported sharing with HCPs, 60% share with family and 34% with friends.
Bauer et al., 2017/Patient-oriented health technologies: Patients' perspectives and use	Mobile health data (electronic data)/Primary	Patients (n = 918) who visited one of 6 primary care clinics in the Northwest US. Most participants were females (75%). Average age of 42,7 years (SD = 15.9 years).	June 2013 (2 weeks)/USA	Survey-based study	To investigate primary care patients' comfort sharing health information through mobile devices, and patients' awareness and use of patient portals.	More patients were comfortable sharing mobile health information with providers than having third parties store their information (i.e., companies that are not a part of the provider system) (62% vs 30%, Somers D = 0.33, p < 0.001).
**Studies reporting sharing behavior/attitudes/intentions for primary purposes depending on data type**						
Serrano et al., 2016/Willingness to exchange health information via mobile devices: Findings from a population-based survey	Health information tracked through mobile devices (electronic data)/Primary	3165 patients from the Health Information National Trends Survey (HINTS) 4, cycle 3. About 30% of the respondents were aged 35–49 years, 51% were female, and 67% were non-Hispanic white.	September–December 2013/USA	Survey-based study	To examine willingness to exchange different types of health information via mobile devices and assess whether sociodemographic characteristics and trust in clinicians were associated with willingness in a nationally representative sample.	Compared with diagnostic information, participants were willing the most to exchange appointment reminders (OR = 6.66; 95% CI, 5.68–7.81), general health tips (OR = 2.03; 95% CI, 1.74–2.38), medication reminders (OR = 2.73; 95% CI, 2.35–3.19), laboratory/test results (OR = 1.76; 95% CI, 1.62–1.92), vital signs (OR = 1.63; 95% CI, 1.48–1.80), lifestyle behaviors (OR = 1.40; 95% CI, 1.24–1.58), and symptoms (OR = 1.62; 95% CI, 1.46–1.79).
**Studies reporting sharing behavior/attitudes/intentions for primary purposes depending on data user**						
Wang et al., 2019/Mobile and connected health technology needs for older adults aging in place: Cross-sectional survey study	Mobile and wearable health devices data (electronic data)/Primary	51 participants recruited from a senior community centre, 71% were, with an average age of 70 (SD = 8) years.	2016/Texas, USA	Survey-based study	This study aimed to assess older adults' perceptions of using mobile and connected health technologies.	Participants were mostly interested in sharing different health data from mobile and connected devices (such as heart rate, blood pressure, etc) with their health care providers followed by family, online communities, friends, and no one.
**Studies reporting sharing behavior/attitudes/intentions for primary purposes that reported results other than willingness to share**						
Vervier et al., 2019/“Attitude”- mHealth apps and users' insights: An empirical approach to understand the antecedents of attitudes towards mHealth applications.	Data from mobile health apps (electronic data)/Primary	11 focus group participants; 132 survey participants - 62% were female and 38% were male. The age range was between 18 and 63 years with M = 35.9 (SD = 23.5).	Summer 2017/Germany	Mixed methods study	The aim of the study is to explain the antecedents of the attitude towards mhealth apps and to derive guidelines for digital health care actors.	NR
Abdelhamid et al., 2021/Fitness tracker information and privacy management: Empirical study	Fitness tracker information (electronic data)/Primary	270 participants. Of the 270 participants, 77.8% (n = 211) were male. The majority of the participants were between the age of 25 and 34 years (163 participants).	NR/USA	Scenario-based survey study	This study investigates the influence of granting users' granular privacy control on their willingness to share fitness information.	NR
**Studies reporting sharing behavior/attitudes/intentions for secondary purposes**						
Chen et al., 2016/A Study to Determine the popular lifestyle smartphone applications and willingness of the public to share their personal data for health research	Self-generated health and lifestyle data from smartphone apps and wearable devices (electronic data)/Secondary	101 adults, 83.16% were female, the majority (66.3%) were aged between 18 and 25 years.	October 13, 2014, and March 10, 2015/Sidney, Australia	Survey-based study	The aim of this study was to pilot a survey to explore patterns of behavioral tracking using smartphone lifestyle apps and individuals' willingness to share their app-generated data.	Most participants were willing to share their personal health data for research (77%).
Alaqra and Kane 2020/Wearable devices and measurement data: An empirical study on eHealth and data sharing	Wearable devices data that measured data in general and stress data in the workplace (electronic data)/Secondary	29 participants, 53% (n = 16) were males and 40% (n = 12) females. 50% of the participants were under 40.	2019/Sweden and Ireland	Interview study	This interview study with 29 users of wearable devices reports perspectives on privacy concerns towards sharing of measured data in general, and measured stress data in the workplace.	Sharing measured fitness data is accepted (72%) and sharing stress measured data for the common good (48%), despite privacy concerns.
Cloos et al., 2022/Acceptance of data sharing in smartphone apps from key industries of the digital transformation: A representative population survey for Germany	Self-generated health and lifestyle data from smartphone apps (electronic data)/Secondary	Representative survey for the German population. The final sample included 1013 participants with a female share of 51.73% (n = 524) and an average age of 45.81 years (SD = 14.42).	September 2020/Germany	Vignette study–of the 5 scenarios, 2 were relevant for this review–one focusing on personal, health and nutrition data, with the end user being a health insurance company or the Federal Ministry of Health.	This study investigated the acceptability of data sharing in different vignettes describing five types of these apps from key industries of the digital transformation.	Overall, not a single acceptance value is greater than zero and therefore all values are in the unacceptable range–this refers both to the analysis focusing on different recipients, and on information attributes. There was no significant treatment effect for any of the different recipients and information attributes.
Pilgrim & Bohnet-Joschko 2022/Donating health data to research: Influential characteristics of individuals engaging in self-tracking	Self-tracked health data (electronic data)/Secondary	German health self-trackers (N = 919). Of the 919 participants 68% were women and 32% were men. The most consistent age groups are the ones between 18 and 34 years old (45%) and the one between 35 and 54 (46%).	January and February 2021/Germany	Survey-based study	The objectives of this study are to provide initial insights into the effects of different health data requests and to outline specific user characteristics that seem to influence voluntary data donation willingness among active (Germany) health self-trackers for research positively.	Users' willingness to disclose data as a “donation” more than doubled compared to their “sharing” behavior (willingness to donate = 4.5/10, sharing frequency = 2.09/10).
**Studies reporting sharing behavior/attitudes/intentions for secondary purposes depending on data type**						
Hartmann et al., 2019/Utilization of patient-generated data collected through mobile devices: Insights from a survey on attitudes toward mobile self-monitoring and self-management apps for depression	Data from apps for the self-monitoring and self-management of depression (electronic data)/Secondary	998 participants with a mean age of 38.29 (SD 12.358, range 18–84) years. 67.2% (671/998) were female. Of them, 668 participants have been diagnosed with unipolar depressive disorder at least once in their life and they were included in the analysis.	January 2017–March 2017/Germany	Survey-based study	The aim of this survey was to provide descriptive data upon usage and anticipated usage of self-monitoring and self-management of depression and preferences of potential users in terms of documented parameters and data-sharing options.	Low preferences were found for sharing data with health insurance companies (4.5%, 30/668) and sharing data with friends (12.7%, 85/668). Higher preferences were found for: science (62.4%), psychotherapist (62.4%), GP (44%).
**Studies reporting sharing behavior/attitudes/intentions for secondary purposes that reported results other than willingness to share**						
Heidel et al., 2021/Pricing through health apps generated data—Digital dividend as a game changer: Discrete choice experiment	Self-tracked data - wearable and health app data (electronic data)/Secondary	842 respondents participated in the main survey, and 272 respondents participated in the second survey	December 2019 (3 weeks)/Germany	A discrete choice experiment alternative, a separated adaptive dual response	The objective of this paper is to study under which circumstances wearable and health app users would accept a compensation payment, namely a digital dividend, to share their self-tracked health data.	NR
**Studies reporting sharing behavior/attitudes/intentions for primary and secondary purposes**						
Maus et al., 2021/Privacy personas for IoT-based health research: A privacy calculus approach	Mobile health data (electronic data)/Primary and secondary	85 participants. The majority were aged between 18 and 35 years old. 56% were females (n = 48) and 41% (n = 35) were male.	April 2020/Sweden	Survey-based study	In this study, the authors developed a privacy calculus model adapted for Internet of Things -based health research using citizen science for user engagement and data collection.	67% (n = 57) were willing to share data like location, steps, calories, and heart rate in medical studies. Respondents felt most comfortable with sharing mobile health data with clinicians and doctors (75%), public health institutions (60%), and research centres or universities (71%), then for a private company (11%) or non-profit company (35%).
Woldaregay et al., 2020/User expectations and willingness to share self-collected health data	Self-collected health data from wearable devices and mobile health apps (electronic data)/Primary and secondary	447 adult participants. Sixty-one (61) participants had diabetes, 82 participants had another chronic disease, and 285 had no chronic disease.	November 2018–August 2019/The majority of participants came from Switzerland (187), Norway (59), US/UK/Australia/Canada (77), France (26), and Germany (13).	Survey-based study	The purpose of this study is to survey and examine factors that may motivate sharing self-collected health data.	Groups do not differ in sharing lifestyle/dietary information, signs of infection, daily mood, geographical location, sleep duration, social environment.

Abbreviations: HD, health data; NR, not reported; AOR, adjusted odds ratio; SD, standard deviation; CI, confidence intervals; M, mean; HCP, healthcare professionals; OR, odds ratio.

**Table 2:** Summary characteristics of studies reporting findings related to sharing attitudes towards sharing of personal health data/information.

Reference/Title	Type of HD/Purpose for data collection (primary vs secondary)	Study population	Duration/period of the recruitment for study sample/Geographical location	Study design	Aim	Attitudes/behavior/willingness towards sharing HD
**Studies reporting sharing behavior/attitudes/intentions for primary purposes**						
Trachtenbarg et al., 2017/The benefits, risks and costs of privacy: patient preferences and willingness to pay	PHID/Primary	834 patients attending physician offices at 14 sites. The average patient age was 50.2 (±15.7) years. Most patients were female (62%), white (91%).	NR/USA	Survey-based study	To: (1) compare medical privacy preferences to privacy preferences in other areas; (2) measure willingness to pay the cost of additional privacy measures; and (3) measure willingness to accept the risks of not sharing information.	Over 95% of patients were willing to share all their medical information with their treating physicians. There was no difference in willingness to share between primary care and specialty sites including psychiatry and an HIV clinic.
Medford–Davis et al., 2017/Health information exchange: What do patients want?	PHID from EHRs (electronic data)/Primary	982 adult patients presenting to an ED. The median age of participants was 53 (IQR: 35.5–64), 57.06% were female.	28 April and 11 August 2015/USA	Questionnaire-based interview study	To determine whether ED patients want to share their medical records across health systems through HIE and if so, whether they prefer to sign consent or share their records automatically.	92.3% were willing to share their data in a HIE. Of those who were willing to share their data in a HIE, 54.3% wanted to sign consent but 90% of those would waive consent in the case of an emergency.
Yaraghi et al., 2015/Drivers of information disclosure on health information exchange platforms: insights from an exploratory empirical study	PHID for HIE (electronic data)/Primary	20,076 patients in Western New York. Female patients constituted the majority (68.2%) of the population.	June 2008 and August 2011/USA	Dataset consisting of consent choices provided by HEALTHeLINK that provides fully operational query based HIE services to over 3300 healthcare providers.	The objective of this research is to empirically explore the drivers of patients' consent to sharing of their medical records on HIE platforms.	The overall consent rate was 89.9%.
Patel et al., 2012/Consumer Support for health information exchange and personal health records: A regional health information organization survey.	PHID from PHRs (electronic data)/Primary	117 consumers, 73% were female, those aged between 45 and 54 years were presented the most (28%).	August 2008/USA	Survey-based study	The aim of the study was to characterize consumer support for electronic HIE and PHRs in a community where HIE is underway.	Eighty-three percent of respondents (n = 114) reported that they supported medical records being shared electronically between doctors, providers, emergency rooms and other places they received care.
Drake et al., 2022/The relationship of socio-demographic factors and patient attitudes to connected health technologies: A survey of stroke survivors	PHID from EHRs (electronic data)/Primary	80 stroke survivors. Male and Female were almost the same number (respectively M = 40 F = 39). Most respondents (51.3%) were aged between 55 and 74 years.	November–15 December 2020 (6 weeks)/UK	Survey-based study	This paper describes the findings of a survey designed to explore the relevance of socio-demographic factors to attitudes towards connected health technologies in a community of patients.	About three quarters indicated they would be willing to share data in relevant ways. 64/80 said that they would use the CONSULT system to send data to their GP or carer; further, they would be willing to make their health records and sensor data available to the CONSULT system (68/80).
Ancker et al., 2012/Consumer perceptions of electronic health information exchange	PHID for HIE (electronic data)/Primary	800 adult New York State residents. Respondents had a mean age of 49.5 years. Fifty-two percent were female, 74% were white, and 11% Hispanic.	2011/USA	Survey-based study	The goal of this study was to assess consumer perceptions of HIE in a state (New York) with a 6-year history of successful HIE organizations.	Of 800 participants, 69% supported sharing their medical information electronically between doctors, hospitals, and other places they receive care.
Nong et al., 2022/Discrimination, trust, and withholding information from providers: Implications for missing data and inequity	PHID/Primary	US adults (n = 2029)	May 2019/USA	Survey-based study	This study assessed the relationship between withholding information from providers, experiences of discrimination, and multiple types of patient trust.	27.5% of respondents reported ever withholding information from healthcare providers.
Grando et al., 2017/A study to elicit behavioral health patients' and providers' opinions on health records consent	PHID from EHRs (electronic data)/Primary	Fifty patients receiving behavioral healthcare were recruited. 84% were female, and 28% were aged 21–30 years.	NR/USA	Survey-based study	To explore behavioral health patient preferences regarding what health information should be shared for care and whether these preferences vary based on the sensitivity of health information and/or the type of provider involved.	58% of the participants agreed with the board consent (share all or none PHI). Study participants preferred sharing all their health information with primary physicians and behavioral health providers, and less with specialty care providers, pharmacists, and nurses.
Kim et al., 2017/Factors affecting willingness to share electronic health data among California consumers	PHID from EMRs (electronic data)/Primary	800 Californians, 53% were female, 72% were aged between 18 and 64 years.	January 22–February 23, 2013/USA	Survey-based study	The objective of the study is to explore factors that affect consumers' willingness to share electronic health information for healthcare and research.	56.4% would consent to sharing PHI for healthcare.
Dimitropoulos et al., 2011/Public attitudes toward health information exchange: Perceived benefits and concerns	PHID for HIE (electronic data)/Primary	English-speaking adults (n = 1847). Twenty-five percent of the respondents were between 18 and 39 years old, 46% were between 40 and 64 years old, and 29% were 65 years or older. Most respondents were white (60% vs 72% US) or female (59%).	August and November of 2010/USA	Survey-based study	To characterize consumers' attitudes regarding the perceived benefits of electronic HIE, potential HIE privacy and security concerns, and to analyze the intersection of these concerns with perceived benefits.	About half (49%) of respondents reported that they would be willing to share all their health information with their healthcare provider, less than a third (32%) would share some of it, and about 12% would refuse to share any information.
**Studies reporting sharing behavior/attitudes/intentions for primary purposes depending on data user**						
Teixeira et al., 2011/HIV patients' willingness to share personal health information electronically	PHID (electronic data)/Primary	93 people living with HIV/AIDS. 45% of participants were female. 86% were over 40 years old.	NR/USA	Survey-based study	To assess the attitudes of persons living with HIV/AIDS towards having their PHI stored and shared electronically.	The majority (84%) of individuals were willing to share their PHI with clinicians involved in their care. Fewer individuals (39%) were as willing to share with non-clinical staff.
Soni et al., 2019/Perceptions and Preferences About Granular Data Sharing and Privacy of Behavioral Health Patients	PHID from EHRs (electronic data)/Primary	86 behavioral health patients, 68.6% were female.	NR/USA	Survey-based study	To know about data sharing preferences for care and research of behavioral health patients.	Participants were most willing to share their health information with the behavioral providers at the study sites, followed by emergency providers, other non-behavioral providers at the study sites (e.g., primary and specialty care providers, pharmacists), behavioral providers outside the sites, and lastly with other non-behavioral providers outside the study sites.
Pedersen et al., 2015/A cross-sectional survey exploring attitudes towards provincial electronic health record implementation among clients attending the Provincial Sexually Transmitted Infections Clinic in British Columbia	PHID from EHRs (electronic data)/Primary	1004 clients attending a Sexually Transmitted Infections (STI) clinic in Vancouver. The majority (61.3%) of participants were male, while the most representative age groups are 19–29 (40%) and 30–39 (35.5%).	July 2012–March 2013/Canada	Survey-based study	To assess attitudes towards sharing of personal health information through a provincial EHR.	40.6% find unacceptable that other health professionals have access to their clinical records from the STI clinic, including family physician, pharmacist (69.7%), medical specialist (45.1%), nurses in other STI clinics (41.8%) and public health nurses (36.2%).
Kimura et al., 2014/A Survey aimed at general citizens of the US and Japan about their attitudes toward electronic medical data Handling	PHID from EMRs (electronic data)/Primary	Members of the general population: US (n = 200, 57.5% were female, 40% were above 60 years old) and Japan (n = 457, 23.9% were female, 42.5% were above 60 years old)	16–31 October 2007 (Japan), 28 September 2009 (USA)/USA and Japan	Survey-based study	To clarify the views of the general population of two countries (US and Japan), concerning the handling of their medical records electronically.	US: sharing their identifiable EMRs should be limited to the doctors-in-charge and specified doctors referred to by their own doctors. Japan: doctors of the same hospital can share their EMRs.
Kimura et al., 2022/Surveys aimed at general citizens of the US and Japan about their attitudes toward electronic medical data handling - 10 years change, before and after Covid-19	PHID from EMRs (electronic data)/Primary	USA (2022): n = 200, age categories were normally distributed (between 10 and 20% each), 52% were women. Japan (2017): n = 225, 63% were older than 60 years, 73% were men.	2008 & 2022 (USA), and 2007 & 2017 (Japan)/USA and Japan	Survey-based study/Note–the results presented here are only from the 2022 and 2017 surveys.	To clarify the views of the general population of two countries (US and Japan), concerning handling of their medical records electronically, disclosure of the name of disease, secondary usage of information.	Positive answers regarding sharing identifiable EMR data for treating participants' illness to other doctors (in the same/other department, same/other hospital, other, etc.) varied from 35 to 50% for the USA, and 23–77% for Japan.
**Studies reporting sharing behavior/attitudes/intentions for primary purposes depending on other factors**						
Soni et al., 2020/State of the art and a mixed-method personalized approach to assess patient perceptions on medical record sharing and sensitivity	PHID from EHRs (electronic data)/Primary	25 patients with behavioral health conditions, English and Spanish-speaking, 60% were female (60%)	2009 and 2019/USA	Mixed method study	This study designed and piloted a mixed-method approach that employs an individual's own records to assess content sensitivity and preferences for granular data sharing for care and research.	All participants would share all or some of their EHRs data with providers outside the study sites. Twelve (48.0%) wanted to share all data with all providers. Hypothetically, in case of emergency, 89.1% of them would share the most and 72% all the EHR data.
Dhopeshwarkar et al., 2012/Health care Consumers' preferences around health information exchange	PHID for HIE (electronic data)/Primary	Residents in the Hudson Valley of New York State (n = 170). Age within this group varied, with 36% of respondents reporting they were aged between 25 and 44 years, and 40% were aged between 45 and 64 years. Most respondents were white (81%) and 54% were women.	January–April 2008/USA	Survey-based study	To better understand consumer preferences regarding the privacy and security of HIE.	Respondents were generally comfortable with the 3 HIE architecture models: on an editable and readable portable device (83%), at different locations and shared over a secure connection (79%), on a single, central database and shared over a secure connection with the use of a password (68%),
Kim et al., 2015/Comparison of consumers' views on electronic data sharing for healthcare and research	PHID for HIE (electronic data)/Primary	800 Californians, 53% were female, 72% were aged between 18 and 64 years.	January 22–February 23, 2013/USA	Survey-based study	To explore California consumers' views of data sharing for healthcare and research together.	There were three options for PHI sharing for HIE based on the type of consent–11% selected opt-out, 23% preferred opt-in alone and 66% preferred opt-in with “break the glass”. Respondents were most likely to consent if asked by a hospital and least likely if asked by an insurance company.
Esmaeilzadeh 2020/Patients' perceptions of different information exchange mechanisms: An exploratory study in the United States	PHID for HIE (electronic data)/Primary	General public (n = 1624). 54.87% were male, and 45.13% were female. The age range was normally distributed.	July 2020/USA	Survey-based study	This study aimed to shed more light on public perspectives (benefits, concerns, and risks) associated with the four data exchange practices in the health care sector.	42.70% of respondents would more likely share their PHI with health care providers that implemented and utilised a direct exchange mechanism.
**Studies reporting sharing behavior/attitudes/intentions for primary purposes that reported results other than willingness to share**						
Lott et al., 2019/Trust and privacy: How patient trust in providers is related to privacy behaviors and attitudes	PHID (electronic data)/Primary	US general population (n = 542), 51.6% were female, 84.6% were white, the most presented were those aged more than 60 years (26.8%).	2014/USA	Survey-based study	To analyze national survey data to determine how dimensions of patient trust in physicians are related to patient disclosure of health information and to attitudes about information sharing within health care.	NR
Abdelhamid et al., 2017/Putting the focus back on the patient: how privacy concerns affect personal health information sharing intentions	PHID (electronic data)/Primary	1600+ participants using the Health Information National Trends Survey (HINTS). Of the total, 61.39% were (986/1606) females. The average age was about 54 (SD = 16) years.	Data collected in 2014/USA	Survey-based study	This study investigated the factors that influence individuals' intentions to share their PHI electronically with health care providers.	NR
Esmaeilzadeh 2020/The impacts of the privacy policy on individual trust in health information exchanges (HIEs)	PHID for HIE (electronic data)/Primary	1624 participants; most respondents were male (54.5 percent), were white (74.8 percent). Most of the participants (71.6%) were aged between 20 and 39 years.	June 2018/USA	Survey-based study	This study analyzes the effects of cognitive trust and emotional trust on the intention to opt in to HIEs and willingness to disclose health information.	NR
Hwang et al., 2012/The differing privacy concerns regarding exchanging electronic medical records of internet users in Taiwan	PHID for HIE (electronic data)/Primary	213 participants, 50.7% were men, 45.5% were younger than 30 years.	NR/China	Survey-based study	This study explores whether Internet users have different privacy concerns regarding the information contained in EMRs according to gender, age, occupation, education, and EMR awareness.	NR
Holderried et al., 2023/The potential of eHealth for cancer patients-does COVID-19 pandemic change the attitude towards use of telemedicine services?	PHID (via eHealth) (electronic data)/Primary	280 patients underwent outpatient chemotherapy treatment or post-chemotherapy after care. 52% were men, 75% were older than 54 years.	September 2019 and February 2021 excluding March 2020 and July 2020 period due to COVID/Germany	Survey-based study	This study investigates the attitude of cancer patients towards eHealth and the potential impact of COVID-19 on its use.	NR
**Studies reporting sharing behavior/attitudes/intentions for secondary purposes**						
Courbier et al., 2019/Share and protect our health data: an evidence-based approach to rare disease patients' perspectives on data sharing and data protection - quantitative survey and recommendations	PHID/Secondary	Rare disease patients (n = 2013). 76% were women, the majority (49%) were aged between 35 and 49 years.	March–May 2018/88% of the respondents resided in the European Union	International survey-based study	To explore patient and family perspectives on data sharing and data protection in research and healthcare settings.	Almost all respondents would be willing to make their own health data or that of the person they care for available for research purposes, whether it is used to develop new treatments (97%), to improve research on diagnosis (97%) and/or to better understand mechanisms and causes of the disease (97%).
Mursaleen et al., 2017/Attitudes Towards data collection, ownership and sharing among patients with Parkinson's disease	PHID/Secondary	310 patients with Parkinson's disease. Most respondents (208/306) were aged between 55 and 74 years. There was a roughly even split between male and female respondents (45% and 55% respectively, n = 306).	June 2016–September 2016. Respondents (n = 306) were predominantly UK (31%), USA (27%) and Canada (21%) based but, in total, responses covered 20 countries and 5 continents.	Survey-based study	The objective of this paper is to establish patient attitudes to ownership of their own medical data and the sharing thereof.	Although 93% of respondents were willing to share data, only 41% were currently doing so and a further 8% did not know whether they were sharing any information in this way.
Buckley et al., 2011/Public attitudes to the use in research of personal health information from general practitioners' records: a survey of the Irish general public	PHID from medical records/Secondary	1575 adult recipients are nationally representative in terms of age bands, socioeconomic status, and region. 71.6% were women, 26.3% were aged between 46 and 55 years.	NR/Ireland	Mixed methods study	This study explores attitudes among a large sample of the Irish public towards the use of general practice medical record data for research.	89.5% said they would agree to ongoing consent arrangements, allowing the sharing by GPs of anonymous personal health information with researchers without the need for consent on a study-by-study basis.
Weitzman et al., 2010/Sharing medical data for health research: the early personal health record experience	PHID from PCHRs (electronic data)/Secondary	151 early adopters of a live PCHR. Mean age 54 (DS 18). Female 53%.	NR/USA	Mixed method study	The objective was to characterize consumer willingness to share PCHR data for health research and the conditions and contexts bearing on willingness to share.	138 out of 151 (91%) were willing to share medical information for health research.
Muller et al., 2022/Patients' and publics' preferences for data-intensive health research governance: survey study	PHID/Secondary	987 respondents, 58.9% were male, 66% were older than 61	February 9, 2021–May 10, 2021/Belgium, Finland, Germany, Ireland, Netherlands, Portugal, Sweden, UK	Survey-based study	This study aims to inform efforts to design governance frameworks for data-intensive health research, by gaining insight into the preferences of patients and publics for governance policies and measures.	A total of 86.5% (848/981) was in favour of sharing their health data for research purposes.
Tosoni et al., 2021/The use of personal health information outside the circle of care: consent preferences of patients from an academic health care institution	PHID/Secondary	222 patients from an academic health care institute, 59% were aged between 50 and 74 years, 50% were male.	December 2019/Canada	Survey-based study	To acquire an in-depth understanding of the contemporary and specific consent needs of cancer patients at a large academic hospital.	Overall, 83% were willing to share their health information with their own institutional University Health Network researchers.
Habich-Sobiegalla and Kostka 2021/Sharing is caring: willingness to share personal data through contact tracing apps in China, Germany, and the US	PHID/Secondary	Respondents from China, Germany, USA (n = 6464), out of which 51.3% were male. Median in years = 36.	June 5, 2020, and June 19, 2020/China, Germany, and the USA	Survey-based study	To study citizens' willingness to share personal data through COVID-19 contact tracing apps (CTAs).	78% are willing to share health information through CTAs in China, and 41% of respondents were willing in both Germany and the USA.
Weng et al., 2019/Crowdsourcing public opinion for sharing medical records for the advancement of science	PHID from EMRs (electronic data)/Secondary	Amazon Mechanical Turk workers (n = 1774). 58.1% were male and 84.2% were younger than 40.	NR/USA	Survey-based study	This study used Amazon Mechanical Turk to crowdsource public opinions about sharing medical records for clinical research.	More than 74% were somewhat willing to share de-identified records.
Kim et al., 2017/Factors affecting willingness to share electronic health data among California consumers	PHID from EMRs (electronic data)/Secondary	800 Californians, 53% were female, 72% were aged between 18 and 64 years.	January 22–February 23, 2013/USA	Survey-based study	The objective of the study is to explore factors that affect consumers' willingness to share electronic health information for healthcare and research.	74.8% would consent to share PHI for research.
King et al., 2012/Perspectives of Australian adults about protecting the privacy of their health information in statistical databases	PHID/Secondary	Australian adults (n = 700), 29.71% were aged between 45 and 59 years.	February and December 2006/Australia	Mixed methods study	The aim of this study was to discover the public's attitude and views towards privacy in health care.	73% of the participants confirmed they would provide their health records for medical research.
Krahe et al., 2019/Personal health information in research: Perceived risk, trustworthiness and opinions from patients attending a tertiary healthcare facility	PHID/Secondary	249 participants who were attending public tertiary healthcare, 59.84% were female, 33.39% were aged between 45 and 64 years and 40.56% were older than 65 years.	16 October and 3 November 2017/Australia	Questionnaire-based design	To explore the opinions, perceived risks and trustworthiness regarding the use of PHI for research, in a sample of the public attending a tertiary healthcare. facility.	Overall participants were mostly willing to share their PHI (68%).
Lysaght et al., 2021/Trust and trade-offs in sharing data for precision medicine: A national survey of Singapore	PHID/Secondary	1000 respondents, 51.9% were women, the age categories were similarly distributed	2019/Singapore	Survey-based study	The aim of the research was to measure the priorities and preferences of Singaporeans for sharing health-related data for precision medicine.	A total of 64% were generally willing to share de-identified health data for IRB-approved research without re-consent for each study.
Bouras et al., 2020/Non-hispanic white mothers' willingness to share personal health data with researchers: Survey results from an opt-in panel	PHID from EMRs (electronic data)/Secondary	622 healthy non-Hispanic white mothers raising healthy children. Most of the mothers were married (485/622, 78.0%) women aged between 18 and 49 years (444/622, 71.4%).	NR/USA	Survey-based study	This study aimed to assess healthy non-Hispanic white mothers' attitudes in five areas: motivation to share data, concern with data use, desire to keep health information anonymous, use of patient portal and willingness to share anonymous data with researchers.	36.0% (224/622) of mothers were less willing to share their medical record data with researchers.
**Studies reporting sharing behavior/attitudes/intentions for secondary purposes depending on data user**						
Aggarwal et al., 2021/Patient perceptions on data sharing and applying artificial intelligence to health care data: Cross-sectional survey	PHID/Secondary	Patients from a large multisite teaching hospital in the UK (n = 408). 48.5 were women (9% did not answer), the majority (30.15%) were aged between 46 and 64 years, and the majority (42.6%) were white/British.	June 2018–August 2018 (12 weeks)/UK	Cross-sectional survey	This study aimed to identify current awareness regarding health data research, and for obtaining opinions and views on data sharing for AI research purposes, and on the use of AI technology on health care data.	Most were comfortable with sharing health data with the National Health Service (318/408, 77.9%) or universities (268/408, 65.7%), but far fewer with commercial organizations such as technology companies (108/408, 26.4%).
Velarde et al., 2021/Citizens' views on sharing their health data: the role of competence, reliability and pursuing the common good	PHID/Secondary	73 adult participants, aged between 18 and 78 years old, with 46 women and 27 men.	NR/Switzerland	Mixed method study	To address questions regarding how people consider what they do or do not consent to and the reasons why.	Around 92% of people replied they would donate data for disease research to the public hospital. Other entities such as the pharmaceutical industry, public services, other private companies, and patient organizations in general received low support.
Kim et al., 2015/Comparison of consumers' views on electronic data sharing for healthcare and research	PHID (electronic data)/Secondary	800 Californians, 53% were female, 72% were aged between 18 and 64 years.	January 22–February 23, 2013/USA	Survey-based study	To explore California consumers' views of data sharing for healthcare and research together.	Respondents were more likely to agree to share deidentified information for research than to share identified information for healthcare (76.2% vs 57.3%, p < 0.001). Willingness to share PHI varied depending on the type of research organization - the highest was in case of a hospital (85.5%) and lowest in case of insurance companies (38%).
Kaufman et al., 2016/A Survey of U.S adults' opinions about conduct of a nationwide precision medicine initiative® cohort study of genes and environment	PHID and biospecimen (electronic data)/Secondary	People were contacted by GfK (n = 2601), 52% were women, 55% were White, 32% were aged between 45 and 59 years and 33% above 60 years.	May 28, 2015, and June 9, 2015/USA	Survey-based study–the influence of different consent models on willingness to share data was examined by randomizing participants to one of eight consent scenarios.	To measure attitudes about and inform the design of the Precision Medicine Initiative's planned national cohort study.	Willingness to share PHI and samples depending on the consent model: the study-by-study (72%), menu (75%), and dynamic consent models (73%) while 64% would share with the study under the broad consent model. Willingness depending on the type of user: researchers at the NIH (79%) and U.S. academic researchers (71%), “pharmaceutical or drug company researchers” (52%) or “other government researchers” (44%).
Soni et al., 2020/State of the art and a mixed-method personalized approach to assess patient perceptions on medical record sharing and sensitivity	PHID from EHRs (electronic data)/Secondary	25 patients with behavioral health conditions, English and Spanish-speaking, 60% were female (60%)	2009 and 2019/USA	Mixed method study	This study designed and piloted a mixed-method approach that employs an individual's own records to assess content sensitivity and preferences for granular data sharing for care and research.	Most (76.0%) participants were extremely willing to share research conducted by study sites and universities (64.0%). 52.0% of the participants showed willingness to share with non-profit organizations, 48% with government agencies and 40% with pharmaceutical companies.
Soni et al., 2019/Perceptions and Preferences About Granular Data Sharing and Privacy of Behavioral Health Patients	PHID from EHRs (electronic data)/Secondary	86 behavioral health patients, 68.6% were female.	NR/USA	Survey-based study	To know about data sharing preferences for care and research of behavioral health patients.	Most participants (96.5%) indicated they would be extremely to somewhat willing to share their data for research with their care facilities and universities, less with drug development companies and government agencies.
Grando et al., 2017/A study to elicit behavioral health patients' and providers' opinions on health records consent	PHID from EHRs (electronic data)/Secondary	Fifty patients receiving behavioral healthcare were recruited. 84% were female, and 28% were aged 21–30 years.	NR/USA	Survey-based study	To examine behavioral health patients' willingness to share PHI for research purposes.	Participants would share their PHI the most for research investigating the conditions they have (61%) and for research done by non-profit organizations (38.3%), and least if the research is done by government agencies (21.3%).
Grande et al., 2013/Public preferences about secondary uses of electronic health information	PHID from EHRs (electronic data)/Secondary	3064 adults (Hispanic, non-Hispanic African American, and non-Hispanic white). 49.8% were women, 65% were older than 40 years.	November 9, 2012–December 2, 2012/USA	Survey based study with an embedded conjoint experiment	To measure patient preferences about sharing their electronic health information for secondary purposes.	In unadjusted linear regression models, marketing uses (β = −1.55), quality improvement uses (β = −0.51), drug company users (β = −0.80), and public health department users (β = −0.52) were associated with less willingness to share health information than research uses and university hospital users (all p < 0 0.001).
Jung et al., 2020/Individual willingness to share personal health information with secondary information users in South Korea	PHID from EHRs (electronic data)/Secondary	Members of online health communities (n = 104)	NR/South Korea	Survey-based study	To investigate individuals' willingness to share their health information based on anonymity, information type (partial vs whole), and the type of information user.	The overall mean value of the willingness was 3.5 on the 7-point Likert scale. Willingness to share was higher for health-related governmental agencies and health professionals than for other governmental agencies.
Kim et al., 2017/iCONCUR: informed consent for clinical data and bio-sample use for research	PHID (electronic data)/Secondary	Patients from 4 outpatient clinics (n = 394). However, 126 actively logged in to the website (84 from HIV clinic, and 42 from internal medicine clinics). 71.14% were male.	August 2014–August 2015/USA	Experimental study that contained a web-based tiered informed consent tool	This study developed, implemented, and evaluated the feasibility of a secure, tiered e-consent web service designed to elicit and honor data sharing preferences in an academic medical centre data delivery system for research.	The majority consented to share most of their data and specimens with researchers. The number of items declined was higher for for-profit institution recipients. Most respondents (79%) indicated that they would be equally willing to share their data for research and for health care.
f	PHID/Secondary	2537 individuals residing in Australia. 50.65% were women, 34.41% were aged between 30 and 49 years.	May 17, 2019–June 7, 2019/Australia	Survey-based study	This study aims to explore public attitudes in Australia toward sharing government health data with the private sector.	Between 51.8% and 57.98% of all participants were willing to share their data, with slightly fewer in favour of sharing to improve health services (51.99%) and a slightly higher proportion in favour of sharing for research and development (57.98%).
Karampela et al., 2019/Connected health user willingness to share personal health data: Questionnaire study	PHID/Secondary	8004 people using connected health services across four European countries, 50% were female, aged between 18 and 65 years.	December 2018/Finland, the Netherlands, Germany, and France	Survey-based study	To explore user attitudes toward sharing personal health data.	22.63% of users are willing to share their personal PHI for scientific research, 29.78% would not share at all, 14.23% would for financial compensation, 11.86% would for public health interests.
Ziefle and Valdez, 2018/Decisions about medical data disclosure in the internet: An age perspective	PHID/Secondary	173 adult participants, 50.3% were male respondents. The age range was wide, with participants from 18 to 65 years of age (M = 43.5 years, SD = 12.8).	NR/Germany	Focus groups were followed by a conjoint-decision study	The aim of this study was to investigate acceptance-relevant criteria that people apply to the vision of sharing their medical data on the Internet.	Independently disliked of their age, users disagreed to sharing data regarding mental illnesses, also disliked high identification risks and commercial use of the data but would be willing to share data scientific purposes.
Johansson et al., 2021/Preferences of the public for sharing health data: Discrete choice experiment	PHID (electronic data)/Secondary	5199 adult respondents. Mean ages of the respondents were 50.4 years (SD 16.9) in Sweden, 48.3 years (SD = 17.2) in Norway, 49.9 years (SD = 15.9) in UK. 48.2 years in (SD = 17.2) in Iceland. Most equal number between man and women in all the countries examined.	August–November 2020/The UK, Norway, Iceland, and Sweden	Discrete choice experiment	The aim of this study is to elicit the preferences of the public in different Northern European countries for sharing health information in different contexts.	Respondents in this study indicated that they preferred to share their data when a national authority was going to be the new user of the data. The second preferred new user was an academic research project.
**Studies reporting sharing behavior/attitudes/intentions for secondary purposes depending on data type**						
Kimura et al., 2022/Surveys aimed at general citizens of the US and Japan about their attitudes toward electronic medical data handling - 10 years change, before and after covid-19	PHID from EMRs (electronic data)/Secondary	USA (2022): n = 200, age categories were normally distributed (between 10 and 20% each), 52% were women. Japan (2017): n = 225, 63% were older than 60 years, 73% were men.	2008 & 2022 (US), and 2007 & 2017 (Japan)/USA and Japan	Survey-based study/Note–the results presented here are only from the 2022 and 2017 surveys.	To clarify the views of the general population of two countries (US and Japan), concerning handling of their medical records electronically, disclosure of the name of disease, secondary usage of information.	Depending on the type of health data, positive answers regarding sharing unidentified EMR data and samples for research purposes was between 42 and 48% for the USA and between 41 and 65% for Japan.
Nunes et al., 2021/Public attitudes to digital health research repositories: Cross-sectional international survey	PHID/Secondary	1600 respondents; Most of the sample is aged between 18 and 27 years (933/1600, 58.31%); 55.69% were females.	March 2020–December 2020/Denmark and Brazil	Survey-based study	This study investigates public attitudes toward possibly contributing to digital health research repositories to identify factors for their acceptance and to inform future developments.	Most participants feel comfortable or very comfortable sharing: food consumption (84.63%), alcohol consumption (79.63%), physical illness diagnosis (77.38%), physical activity levels (75.94%), mental illness diagnosis (66.25%), blood samples (64.31%), DNA samples (46.88%),
Garret and Young 2022/Ethical views on sharing digital data for public health surveillance: Analysis of survey data among patients	PHID from EHRs and social media data (electronic data)/Secondary	One hundred and sixty-one participants with medical conditions, out of which 60.2% were female.	March 2020/USA	Survey-based study	This study aims to examine participants' social media use and comfort sharing their data with health researchers.	More than one third of participants reported being very comfortable sharing electronic health data and social media data for personalized healthcare and to help others–39.8% for sharing EHRs, and 34.2% for sharing social media data.
**Studies reporting sharing behavior/attitudes/intentions for secondary purposes depending on other factors**						
Tosoni et al., 2022/Patient consent preferences on sharing personal health information during the COVID-19 pandemic: “the more informed we are, the more likely we are to help”	PHID/Secondary	183 patients were included in the pandemic cohort and 222 in the pre-pandemic cohort; all were patients of a large Canadian cancer centre.	2019/Canada	Survey-based study	The study sought to ascertain whether there were differences in consent preferences between pre-pandemic times compared to during Wave 1 of the COVID-19 global pandemic, and to better understand the reasons behind these preferences.	Patients in the pandemic cohort were significantly more comfortable with sharing all information and biological samples, sharing information with the health care institution, with researchers at other hospitals, sharing PHI provincially, nationally, and internationally compared to the pre-pandemic cohort.
Corman et al., 2022/Public comprehension of privacy protections applied to health data shared for research: An Australian cross-sectional study	PHID from EHRs (electronic data)/Secondary	317 participants; most of the respondents were aged between 25 and 34 (128/317). 50.9% were women.	Between 27 October and 26 November 2020/Australia	Cross-sectional survey describing a data-sharing scenario motivated by medical research where data could be shared: raw, de-identified, aggregated, and differential privacy applied to aggregated data.	To assess how accurately people understood the effectiveness of techniques for protecting the privacy of shared health data.	There was a big tolerance for researcher use of health data with consistent preference to share data when better privacy-preserving techniques were employed. There was a slight preference for aggregated data over differential privacy, despite differential privacy being objectively more secure.
Grande et al., 2015/Are patients with cancer less willing to share their health information? Privacy, sensitivity, and social purpose	PHID from EMRs and mobile app data (electronic data)/Secondary	Nationally representative participants (n = 3336) with and without prior cancer. The mean age was 60.4 years old (range: 25–91 years). Study participants were predominantly female (81%), white (92%), non-Hispanic (95%).	November 9, 2012–December 2, 2012/USA	Mixed method study (online survey with an embedded conjoint experiment)	Understanding patient views on reuse of health information is essential to shape privacy policies and build trust in these initiatives.	Participants with and without a prior diagnosis of cancer had a similar willingness to share health information (0.27; p = 0.42). Cancer and noncancer respondents rated uses and users similarly.
**Studies reporting sharing behavior/attitudes/intentions for secondary purposes that reported results other than willingness to share**						
Helou et al., 2021/Factors related to personal health data sharing: Data usefulness, sensitivity, and anonymity	PHID/Secondary	112 respondents. The average age was M = 30.32, SD = 10.37.57.1% were women.	First week of December 2020/Middle East, Asia, Europe, North America, Oceania, South America	Survey-based study	To examine the relationships between the perceived usefulness, sensitivity, and anonymity of personal health data and people's willingness to share it with researchers.	NR
Valdez and Ziefle, 2019/The users' perspective on the privacy-utility trade-offs in health recommender systems	PHID/Secondary	Study 1: n = 281, 54% were female, mean age 39.7 (14.3). Study 2: n = 243, 51% were female, mean age 49.6 (12.3).	May 2016/Germany	Two conjoint-decision studies	To investigate importance and utility of privacy-preserving techniques related to sharing of personal health data for k-anonymity and differential privacy.	NR
Grande et al., 2014/The importance of purpose: moving beyond consent in the societal use of personal health information	PHID from EHRs (electronic data)/Secondary	3064 adults (Hispanic, non-Hispanic African American, and non-Hispanic white). 49.8% were women, 65% were older than 40 years.	November 9, 2012–December 2, 2012/USA	National experimental survey using vignettes	Examine public support for secondary uses of electronic health information under different consent arrangements.	NR
**Studies reporting sharing behavior/attitudes/intentions for primary and secondary purposes**						
Fylan and Fylan 2021/Co-creating social license for sharing health and care data	PHID/Primary and secondary	General public from the survey (n = 1031)–63% were female, respondents were mainly white (91%); a range of ages was included: 14% were 18–24, 26% were 25–44, 39% were 45–64, and 21% were over 65.	NR/England	Sequential mixed methods approach comprising focus groups, surveys, and co-design groups	This study explores the public's willingness to contribute data for direct care and research purposes, how the concerns they have differ between data sharing for different reasons, and the principles that should apply when their data are used for different purposes.	Survey respondents agreed that their GP (98%) and hospital doctors and nurses (93%) should be able to see their health and care records. There was more limited support for pharmacists (37%), care staff (36%), social workers (24%) and researchers (24%).
Trinidad et al., 2020/The public's comfort with sharing health data with third-party commercial companies	PHID/Primary and secondary	US public (n = 1841), 49.05% were male. 12% of the participants were under the age of 29, and 31% of participants were over the age of 60.	May 2019–June 2019/USA	Survey-based study	To describe the public's comfort with sharing health data with third-party commercial companies for patient and business purposes and how this comfort is associated with demographic factors, perceived healthcare access, and concerns about privacy.	The public is more comfortable sharing health data with third party commercial companies for patient purposes as compared to business purposes (paired t = 39.84, p < 0.001).
Whiddett et al., 2006/Patients' attitudes towards sharing their health information	PHID/Primary and secondary	Adult primary-care patients (n = 200). 68% female and 56% were aged between 18 and 30.	NR/New Zealand	Survey-based study	To investigate (1) the attitudes of patients toward sharing their medical information and (2) whether patients considered themselves to be well-informed about the uses that are made of their information.	Respondents were generally willing to have their information shared between health professionals, but not with administrators, researchers, or other government departments. Respondents were more prepared to share anonymous and less personal information.
Kim et al., 2019/Older adults' willingness to share their personal and health information when adopting healthcare technology and services	PHID (electronic data)/Primary and secondary	Older adults at local senior centres in South Korea (n = 170; mean age = 77; 45.9% women).	April and May 2017/South Korea	Survey-based study	This study aims to explore factors influencing older adults' willingness to share their PHI when using healthcare technologies and services such as wearable electronic devices and relevant services.	Higher proportions of older adults reported being willing to share their information with family and hospitals than with researchers, government agencies, device developer/corporations, or insurance companies (80.4%, 66.1% vs 39.9%, 23.5%, 25.2%, 19.0%, respectively).
Patil et al., 2016/Public preferences for electronic health data storage, access, and sharing - evidence from a pan-European survey	PHID (electronic data)/Primary and secondary	20,882 respondents, 52.3% were female. All age groups were distributed from 10 to 19%, with those older than 65 years being the most presented (19.1%)	August–November 2013/27 European Union member countries	Survey-based study	To assess the public's preferences regarding potential privacy threats from devices or services storing health-related personal data.	Averse to share health data: with their immediate family home care nurses; Strongly averse: health insurance companies, private sector pharmaceutical companies and academic researchers.
Whiddett et al., 2016/Consent and widespread access to personal health information for the delivery of care: a large-scale telephone survey of consumers' attitudes using vignettes in New Zealand	PHID from EHRs (electronic data)/Primary and secondary	4209 adult participants aged 18+ years, 68.7% were female, 57.6% were aged between 35 and 65 years.	2008/New Zealand	Survey-based vignette study	To examine factors which influence the public's willingness to consent to share their health information in a national EHR.	Rates of consent ranged from 89% (95% CI: 87%–92%) for sharing of information with hospital doctors and nurses to 51% (95% CI: 47%–55%) for government agencies.
Perera et a., 2011/Views on health information sharing and privacy from primary care practices using electronic medical records	PHID from EMRs (electronic data)/Primary and secondary	511 patients (mean age 60.3 years, 49.6% female) And 46 physicians (mean age 47.2 years 37.0% female)	2002–2003/Canada	Survey based study	To determine how patients and physicians balance the perceived benefits and harms of sharing electronic health data for patient care and for secondary purposes.	Primary use: >90% supported the computerized sharing of EMRs among their health care professionals. Secondary use: <70% supported reuse of de-identified HD. 22%, 67%, 45% and 40% of patients would not share EMRs with researchers, private insurance companies, pharmaceutical industry and the government, respectively.
Patel et al., 2011/Low-income, ethnically diverse consumers' perspective on health information exchange and personal health records	PHID from PHRs (electronic data)/Primary and secondary	214 respondents, 58% were aged between 18 and 34 years, 78% were female.	October and November 2008 (6 weeks)/USA	Survey-based study	To survey low-income, ethnically diverse consumers regarding their attitudes towards providers' use of electronic HIE and consumer use of HIE through PHRs.	Willingness to share PHR: with primary care doctors (73%), other doctors or health care providers caring for them (55%), health plans (18%), employers (4%) or government officials (3%), no one (6%).
Weitzman et al., 2012/Willingness to share personal health record data for care improvement and public health: a survey of experienced personal health record users	PHID from PCHRs (electronic data)/Primary and secondary	261 PCHR users who were patients over 18 years old or parents of patients, 21 (8%) were patients and the rest were parents or guardians of patients.	NR/USA	Survey-based study	To characterize consumer willingness and unwillingness (reticence) to share PCHR data across health topics, and with different stakeholders, to advance understanding of this issue.	Of 261 respondents (56% response rate), more reported they would share all information with the state/local public health authority (63.3%) than with an out-of-hospital provider (54.1%) (OR 1.5, 95% CI: 1.1, 1.9; p = 0.005); few would not share any information with these parties (respectively, 7.9% and 5.2%).
Bosanac and Stevanovic 2022/Trust in e-health system and willingness to share personal health data	PHID via e-health (electronic data)/Primary and secondary	102 respondents recruited via KwikSurveys platform (62 F, 35 M, 5 other), average age M = 43.04 (range 25–75), SD = 10.89.	July 2021/Croatia	Survey-based study	To determine familiarity with the e-health system among Croatian citizens, trustfulness in the system itself, and willingness to share PHI with physicians or researchers.	Overall, participants were highly willing to share their health data with their physicians and research, with scores ranging from 7.9 for SD to 9 for allergies.
Bakken et al., 2022/Attitudes of mental health service users toward storage and use of electronic health records	PHID from EHRs (electronic data)/Primary and secondary	253 Norwegian mental health service users. The majority (71%) of participants were women. The most representative age groups were 35–44 years (34%) and 45–54 years (33%).	NR/Norway	A mixed-methods, cross-sectional study	The authors examined service users' awareness, attitudes, and opinions about EHR data storage and sharing.	Most service users (N = 180, 71%) reported that it was ethical to share EHRs for health care and research.
Riordan et al., 2015/Patient and public attitudes towards informed consent models and levels of awareness of Electronic Health Records in the UK	PHID from EHRs (electronic data)/Primary and secondary	3157 adult patients and members of the public in primary and secondary care clinics in West London, UK	1st August 2011 (6 weeks)/UK	Survey-based study	To explore levels of public awareness about EHRs and to examine attitudes towards different consent models with respect to sharing identifiable and de-identified records for healthcare provision, research, and planning.	Most reported that they would expect to be explicitly asked for consent before their identifiable record was accessed (91%). However, half (49%), reported that they would not expect to be asked for permission before their de-identified record was accessed.

Abbreviations: HD, health data; NR, not reported; PHID, personal health information and data; PHI, personal health information; SD, standard deviation; AI, artificial intelligence; AOR, adjusted odds ratio; CI, confidence interval; HIE, health information exchange; EHR, electronic health record; PCHR, personally controlled health records; OR, odds ratio; IQR, interquartile range; ICU, intensive care unit; GP, general practitioner; EMR, electronic medical records; ED, emergency department.

**Table 3:** Summary characteristics of studies reporting findings related to attitudes towards sharing of biobank health data.

Reference/Title	Type of HD/Purpose for data collection (primary vs secondary)	Study population	Duration/period of the recruitment for study sample/Geographical location	Study design	Aim	Attitudes/behavior/willingness towards sharing HD
**Studies reporting sharing behavior/attitudes/intentions for secondary purposes**						
Barnes et al., 2020/Biobanking for genomic and personalized health research: Participant perceptions and preferences	Biobank data/Secondary	196 participants; based on the SpecTRA study 47% of participants are female, with an average age of 69 years, and 90% were white.	NR/Canada	Survey-based study	To examine to explore broader biospecimen and data sharing preferences among a larger group of patients who had opted into a Permission to Contact for research program.	98% said they were willing to provide a sample and/or information to facilitate the project's goals, and 96% to health research in general.
Critchley et al., 2012/Predicting intention to biobank: a national survey	Biobank data/Secondary	1000 Australian adults; mean age was 53.31 years (SD = 16.73; range = 18–94)) and 66% were females.	NR/Australia	Survey-based study	To determine those factors important in determining the public's intention to donate a biological sample to a publicly funded biobank and allow that sample to be linked with medical records.	On average, respondents in this research showed a strong willingness to participate in biobank research (74.5%) and allow their sample to be linked with their medical records (70.3%).
Sanderson et al., 2017/Public attitudes toward consent and data sharing in biobank research: A large multi-site experimental survey in the US	Biobank data/Secondary	13,000 individuals from 11 US healthcare systems in the eMERGE Network. Sixty-three percent of participants were female; 51% were white.	April 2015–July 2015/USA	Experimental survey-based study	The aim of the present study was to examine patients' attitudes toward participating in biobank research using an experimental study design that randomly assigned participants to different consent and data sharing conditions.	66% (95% CI: 63%–69%) of population-weighted respondents stated they would be willing to participate in a biobank; willingness and attitudes did not differ between respondents in the three scenarios.
Antommaria et al., 2018/Parents' attitudes toward consent and data sharing in biobanks: A multisite experimental survey	Biobank data/Secondary	Patients recruited at the 11 eMERGE Network sites (n = 5737), 73.2% were women, 57.2% were aged between 36 and 50 years	April 2015–July 2015/USA	Experimental survey-based study–participants were randomized to receive one of three consent and data-sharing scenarios.	To assess parents' willingness to enroll their children in biobanks, and their perceived benefits, concerns, and information needs under different consent and data-sharing scenarios, and to identify factors associated with willingness.	Overall, 55% (95% CIs: 50–59%) of parents were willing to enroll their youngest minor child in a hypothetical biobank; willingness did not differ between consent and data-sharing scenarios.
Brall et al., 2021/Public willingness to participate in personalized health research and biobanking: A large-scale Swiss survey	Biobank data/Secondary	5156 Swiss residents	16th September 2019 until 31st January 2020/Switzerland	Survey-based study	To explore the Swiss public's willingness, attitudes, and concerns regarding personalized health research participation by providing health information and biological material.	53.6% indicated that they would be willing to participate in a personalized health research project. Respondents were more willing to provide ‘traditional’ types of health data, such as health questionnaires, blood or biological samples, as opposed to social media or app-related data.
Mezinska et al., 2020/Public's awareness of and attitudes towards research biobanks in Latvia: Concerns regarding genetic and medical data donation for research	Biobank data/Secondary	1017 respondents from public in Latvia. Their mean age was 46.3 years (SD = 15.8). Slightly more women than men participated (52.8%).	March 2019/Latvia	Survey-based study	This study analyzed the influence of awareness and attitudes towards biobanks on willingness to participate in biobank studies and on preferred type of informed consent.	Among all participants in the 2019 survey, 36.7% would be willing to provide information about themselves to a biobank, while 53.5% would not be willing to do so.
Ahram et al., 2022/Perceptions, attitudes, and willingness of the public in low- and middle-income countries of the Arab region to participate in biobank research	Biobank data/Secondary	967 participants belonging to the general populations from Egypt (61.3%), Jordan, Morocco, and Sudan. The average age was 33 ± 11 years, the range was 18–73, 56.3% were males.	September 2020–January 2021/Egypt, Jordan, Morocco, and Sudan	Survey-based study	To explore the factors associated with the willingness of the public to participate in biobank research from four low- and middle-income countries in the Arab region.	Participants' willingness to donate biospecimens and health data was less than 10%.

Abbreviations: HD, health data; NR, not reported; eMERGE, the Electronic Medical Records and Genomics (network); SpecTRA, Spectrometry in TIA Rapid Assessment (study); SD, standard deviation.

**Table 4:** Summary characteristics of studies reporting findings related to attitudes towards sharing of genomic and genetic health data.

Reference/Title	Type of HD/Purpose for data collection (primary vs secondary)	Study population	Duration/period of the recruitment for study sample/Geographical location	Study design	Aim	Attitudes/behavior/willingness towards sharing HD
**Studies reporting sharing behavior/attitudes/intentions for primary purposes**						
Riggs et al., 2019/Development of a consent resource for genomic data sharing in the clinical setting	Genomic data/Primary	5162 members of the public	June–July 2016/USA	Survey-based study	To determine whether the consent form and video accurately conveyed key data sharing concepts.	After reading the consent form, 54.3% of the 4865 participants who responded to the question indicated they would consent to broad data sharing.
**Studies reporting sharing behavior/attitudes/intentions for secondary purposes**						
Eikemo et al., 2022/Research based on existing clinical data and biospecimens: a systematic study of patients' opinions	Genomic data (genetic data, health data, and biospecimen)/Secondary	424 consecutive newly discharged hospital patients; 62.5% were female, one third of them were aged between 50 and 69 years.	May 2018–February 2019/Norway	Survey-based study	To investigate newly discharged hospital patients' opinions on secondary use of their hospital data and biospecimens within the context of health research.	90% were positive towards data sharing. Around 90% preferred to be informed (passive consent) or to receive no notification at all for secondary research on their health data and biospecimens. The rest wanted to be asked for active consent. 81% of respondents were positive towards genetic research without active consent.
Mahlmann et al., 2017/Attitudes towards personal genomics and sharing of genetic data among older Swiss adults: A qualitative study	Genomic data/Secondary	40 participants, the mean age of the participants was 71 years ranging from 64 to 92 years. Twenty-one women (52.5%) and 19 men (47.5%).	December 2013 and April 2014/Switzerland	Semi-structured interviews study	To assess the willingness of older Swiss adults to share genetic data for research purposes and associated factors.	22 out of 40 (55%) would explicitly share their data and trust in research. Several factors which might impact the willingness to share data such as sharing data with private companies, generational differences, differences between sharing genetic data or health data, and sharing due to financial incentives.
**Studies reporting sharing behavior/attitudes/intentions for secondary purposes depending on the type of data user**						
Chavarria-Soley et al., 2021/Attitudes of Costa Rican individuals towards donation of personal genetic data for research	Genomic data/Secondary	224 Costa Rican individuals 62.9% female. Age was between 30 and 51.	NR/Costa Rica	Survey-based study	It covers attitudes toward DNA and medical data donation, trust in research professionals and concerns about consequences of reidentification.	Most individuals (89%) are willing to donate their information for research purposes. While most participants were willing to donate information to medical doctors and nonprofit researchers, willingness to donate decreased strongly in the case of for-profit research.
Vidgen et al., 2020/Sharing genomic data from clinical testing with researchers: public survey of expectations of clinical genomic data management in Queensland, Australia	Genomic data/Secondary	1494 adult participants. Participants ranged from 18 to over 75 years of age. Most of them were aged 55 years or more (71.4%, n = 1066), and female (66.8%).	February 2019–May 2019/Australia	Survey-based study	To inform public policy and discussions around genomic data sharing, we sought public opinions on using genomic data contained in medical records for research purposes in the Australian state of Queensland.	Willingness to share genomic data varied from 13.0 (for data being made publicly available) to 91.9% (for sharing with not-for-profit organizations) for anonymous and from 0.9 (for data being made publicly available) to 72.6% (for sharing with universities and research institutes) for identifiable data. Overall, participants were between 12.1 and 31.1% less likely to share their identifiable than anonymous genomic data.
Goodman et al., 2017/De-identified genomic data sharing: the research participant perspective	Genomic data/Secondary	Cancer patients recruited from the Northwest Cancer Genetics Registry (n = 228), their relatives (n = 155), and controls (n = 67).	2013/USA	Survey-based study	This quantitative study set in the USA examines participant preferences and evaluates differences by demographics and cancer history.	Willingness to share was highest for researchers at the same university and non-profit organizations, followed by researchers at other universities in the USA, and lowest for any researcher who requests the information and for-profit, private organizations such as pharmaceutical companies.
Middleton et al., 2020/Global public perceptions of genomic data sharing: What shapes the willingness to donate DNA and health data?	Genomic data/Secondary	36,268 participants from 22 countries. 51.1% were women, age groups were similarly distributed, with those aged 30 years or less being presented the most (24%).	NR/22 countries–Argentina, Australia, Belgium, Brazil, Canada, China, Egypt, France, Germany, India, Italy, Japan, Mexico, Pakistan, Poland, Portugal, Russia, Spain, Sweden, Switzerland, the UK, and the USA	Survey-based study	To explore global public attitudes toward willingness to donate one's DNA and health information to be shared for research (both non-profit and for-profit), together with an under-standing of the factors that shape this.	Willingness to donate one's DNA and health data for research is relatively low, and trust in the process of data being shared with multiple users (e.g., doctors, researchers, governments) is also low. Participants were most willing to donate DNA or health information for research when the recipient was specified as a medical doctor and least willing to donate when the recipient was a for-profit researcher.
**Studies reporting sharing behavior/attitudes/intentions for secondary purposes depending on other factors**						
Parvinen et al., 2023/Exploring the challenges of and solutions to sharing personal genomic data for use in healthcare	Genomic data/Secondary	299 undergraduate students. 67% of the respondents were female, 52% were aged between 18 and 26 years and 48% were aged above 26.	NR/Finland	Survey-based study	To explore how the provision of educational information relates to willingness to consent, as well as differences in privacy concerns, information sensitivity and the perceived trade-off value.	Of the respondents, 65% were initially willing to consent, but after educational information 89% were willing to consent and only 11% remained unwilling to consent.
**Studies reporting sharing behavior/attitudes/intentions for secondary purposes that reported results other than willingness to share**						
Amorim et al., 2022/Benefits and risks of sharing genomic data for research: comparing the views of rare disease patients, informal carers and healthcare professionals	Genomic data/Secondary	159 rare disease patients, 478 informal carers and 63 healthcare professionals	June 2019 and March 2020/Portugal	Survey-based study	To investigate the benefits and risks of sharing genomic data for research, and its associated factors.	NR
Middleton et al., 2019b/Members of the public in the USA, UK, Canada and Australia expressing genetic exceptionalism say they are more willing to donate genomic data	Genomic data/Secondary	General public in the USA, Canada, UK, and Australia (n = 8967). 48.27% were women, 46.15% were aged under 30–40 years.	2016–2018/UK, USA, Canada, and Australia	Cross-sectional, exploratory survey-based study	It examined how acceptance of data sharing pertains to the perceived similarities and differences between DNA and other forms of personal data.	NR
**Studies reporting sharing behavior/attitudes/intentions for primary and secondary purposes**						
Middleton et al., 2019a/Attitudes of publics who are unwilling to donate DNA data for research	Genomic data/Primary and secondary	8967 English-speaking publics from the UK, the USA, Canada, and Australia	NR/UK, the USA, Canada, and Australia	Survey-based study	The objective of the ‘Your DNA, Your Say’ global survey is to explore public attitudes, values, and opinions towards willingness to donate and concerns regarding the donation of one's personal data for use by others.	15.9% of participants (n = 1426) reported that they were unwilling to donate their DNA and medical information to medical doctors, non-profit researchers, or for-profit researchers. A further 16.3% (n = 1462) were unsure in all cases, leaving 67.77% willing to donate their DNA.
Romano et al., 2021/Italian public's views on sharing genetic information and medical information: findings from the ‘Your DNA, Your Say’ study	Genomic data/Primary and secondary	1229 respondents 51% were female, age groups were similarly distributed.	27 August 2019/Italy	Survey-based study	The aim of the study is to gather lay public attitudes toward the access and sharing of DNA information and medical information.	Most (64%) declared they would be willing to share their DNA and medical information for use by at least one data user (doctor, non-profit or for-profit user–data not given for each category).
**Studies reporting sharing behavior/attitudes/intentions for primary and secondary purposes that reported results other than willingness to share**						
Milne et al., 2019/Trust in genomic data sharing among members of the general public in the UK, USA, Canada and Australia	Genomic data/Primary and secondary data	General publics in the USA, Canada, UK, and Australia (n = 8967). 48.27% were women, 46.15% were aged under 30–40 years.	2016–2018/UK, USA, Canada, and Australia	Exploratory survey-based study	This study examined trust in data sharing among the public.	NR

Abbreviations: HD, health data; NR, not reported; OR, odds ratio; DNA, deoxyribonucleic acid.

**Table 5:** Summary characteristics of studies reporting findings related to attitudes towards sharing data categorized as miscellaneous types of data.

Reference/Title	Type of HD/Purpose for data collection (primary vs secondary)	Study population	Duration/period of the recruitment for study sample/Geographical location	Study design	Aim	Attitudes/behavior/willingness towards sharing HD
**Studies reporting sharing behavior/attitudes/intentions for primary purposes**						
Montelius et al., 2008/Individuals appreciate having their medication record on the web: A survey of attitudes to a national pharmacy register	Medication record (electronic data)/Primary	1716 users of the Web-based service “My dispensed medications”.	January 31–March 6, 2007/Sweden	Survey-based study	The aim of the present study was to evaluate the users' attitudes towards their access to “My dispensed medications” as part of a new interactive Internet service on prescribed medications.	Respondents were keener to share their record with a close relative or their physician than with the pharmacy and other health care staff (p < 0.001).
Yu et al., 2021/Perspectives on illness-related stigma and electronically sharing psychiatric health information by people with multiple sclerosis	Psychiatric health information (electronic data)/Primary	3020 participants having multiple sclerosis (MS), 78.8% were female, 48.4% were aged between 45 and 59 years.	NR/USA	Survey-based study	To compare MS patients with and without co-occurring psychiatric diagnoses in terms of their willingness to share their diagnoses and medications through EMRs.	Overall, 96.44% of participants were willing to share their non-psychiatric diagnosis and 87.14% were willing to share their psychiatric diagnosis; 97.70% were willing to share their non-psychiatric medications and 92.78% were willing to share their psychiatric medications.
**Studies reporting sharing behavior/attitudes/intentions for secondary purposes**						
Holm et al., 2021/Control, trust, and the sharing of health information: The limits of trust	Clinical data (mainly electronically stored data)/Secondary	Danish population (n = 994). Of these, 553 (55.6%) were women and 441 (44.4%) men. 51.4% were older than 60 years.	August 2017/Denmark	Survey-based study	To investigate the attitudes of a representative sample of the Danish population towards transfer of clinical data from their general practice for secondary use.	This study found that adult Danes are positive towards research that uses patient data (90% agreed that research using patients' data is important).
Kirkham et al., 2022/Experience of clinical services shapes attitudes to mental health data sharing: findings from a UK-wide survey	Mental health data/Secondary	Diverse sample of UK National Health Service (NHS) users (n = 2187) of which about half (n = 1087) had lifetime experience of mental illness	December 2018–August 2019/UK	Survey-based study	To investigate factors influencing likelihood of sharing these data for research purposes amongst people with and without experience of mental illness.	There was a high level of willingness to share mental (89.7%) and physical (92.8%) health data for research purposes.
Pletscher et al., 2022/Willingness to share anonymised routinely collected clinical health data in Switzerland: a cross-sectional survey	Clinical data/Secondary	General Swiss population (n = 1006) and a population with chronic disease (n = 225)	14 September and 3 October 2020/Switzerland	Survey-based study	The present survey focused on the re-use (secondary use) of hospital-derived health data in anonymized form.	71% of the general population and 81% of the chronic disease group reported that they would share their anonymized health data for medical research.
Savic Kallesoe et al., 2023/Canadians' opinions towards COVID-19 data-sharing: a national cross-sectional survey.	COVID-19 data/Secondary	4981 Canadian participants, 50.9% were female, age groups were similarly distributed	1 March 2022–17 March 2022/Canada	Cross-sectional survey-based study	To inform developments in public health data-sharing in Canada, we explored Canadians' opinions of public health authorities sharing deidentified individual-level COVID-19 data publicly.	79.7% were supportive of provincial/territorial authorities publicly sharing deidentified COVID-19 data, while 20.3% were hesitant/averse/unsure.
Mahmoud et al., 2019/Sharing de-identified medical images electronically for research: A survey of patients' opinion regarding data management	De-identified medical images (electronic data)/Secondary	1083 patients from the Greater Toronto Area attending Sunnybrook Health Sciences Centre for imaging. 52% were female, and age was divided into 7 age groups between 18 years and 75+ and with an overall median age of 60 (IQR = 18, Q1 = 52, Q3 = 70).	2016/Toronto, Canada	Survey-based study	To understand patients' attitudes to sharing their imaging data for research purposes.	76% were willing to share de-identified medical images for research. Most participants gave their family physicians (73%) and other physicians (57%) unconditional data access.
Padrez et al., 2016/Linking social media and medical record data: a study of adults presenting to an academic, urban emergency department	Social media data and emergency medical record data (electronic data)/Secondary	1432 adult Facebook and/or Twitter users seeking care in an urban academic adult ED.	28 March 2014–31 October 2014/USA	Survey-based study	To determine the acceptability to patients and potential utility to researchers of a database linking patients' social media content with their EMR data.	1008 participants (71%) consented to share their social media data for the purposes of comparing it with their EMR.
**Studies reporting sharing behavior/attitudes/intentions for secondary purposes depending on some factors**						
Kongeter et al., 2022/Patients' willingness to provide their clinical data for research purposes and acceptance of different consent models: Findings from a representative survey of patients with cancer	Clinical data/Secondary	Patients with cancer and survivors of cancer (n = 838), 52.6% were men, 30.8% were older than 75 years.	May 2021–July 2021/Germany	Survey-based study	The aim of the study was to learn about patients' attitudes and expectations regarding secondary use of their clinical data.	Willingness was high (96.7%), without any restrictions (62.9%) or under certain conditions (33.8%), independent of the researchers' affiliation (63.4%), and only 22.7% would share with for-profit research companies.
**Studies reporting sharing behavior/attitudes/intentions for primary and secondary purposes**						
Ly et al., 2022/Patient perspectives on the digitization of personal health information in the emergency department: mixed methods study during the COVID-19 pandemic	Emergency medical record data (electronic data)/Primary and secondary	108 participants who had received care in a British Columbia ED within the last 5 years, 71% were female	2019/Canada	Mixed method study	The primary objective was to characterize the views of ED users in British Columbia, Canada, on the impacts of PHI digitization on ED care.	Willingness to share their ED medical record data: with ED staff (up to 90% in emergencies), family physicians (up to 91%), and family caregivers (up to 75%). 73% were willing to share deidentified health data with researchers.

Abbreviations: HD, health data; NR, not reported; IQR, interquartile range; SD, standard deviation; M, mean; EMR, electronic medical records; CI, confidence interval, ED, emergency department.

**Table 6:** Benefits, concerns, and facilitators that were found to impact sharing behavior.

Data type	Primary use	Secondary use
**Person-generated health data**	**Benefits**: Improve health and decrease the risk of disease, injury, or an adverse pregnancy health event (Runkle et al., 2019); Better management of their own condition and improvement of their own health and health of others, receiving emotional support, providing emotional support for others (Brown et al., 2022) **Concerns**: Privacy (Brown et al., 2022; Runkle et al., 2019; Wang et al., 2019); Cost, safety (Wang et al., 2019); To be in social, privacy, psychological risk (Brown et al., 2022); About the relevance and usefulness of their data, feeling their data were irrelevant, lack of adequate data, having limited time to spend with the clinician, privacy concerns, fear of being judged (Luo et al., 2020). **Facilitators** that will increase sharing of fitness information with health care providers: Increased control, negative perceived risk, and perceived benefits (Abdelhamid, 2021).	**Benefits:** To obtain higher quality result, for the common good (Alaqra & Kane, 2020) **Concerns**: Transparency (Runkle et al., 2019); Privacy - those who did not normally share their health-tracking data were more likely than sharers to be concerned about privacy (odds ratio [OR] = 5.93; 95% CI: 2.09–16.78) (Chen et al., 2016) **Facilitators**: Transparency about data processing and no further data sales to third parties, adequate monetary compensation by the pharmaceutical and medical device companies (Heidel et al., 2021);
**Personal health data and information**	**Benefits**: Improve medical care (Ancker et al., 2012; Grando et al., 2017; Patel et al., 2012; Soni et al., 2019); Improve coordination of care (Dimitropoulos et al., 2011; Grando et al., 2017); Improve patient-provider and provider–provider communications (Patel et al., 2012; Soni et al., 2020); Improve safety, avoid duplicate testing (Trachtenbarg et al., 2017); Completeness and accuracy of their records, better control over their HC (Patel et al., 2012). **Concerns**: Privacy (Abdelhamid et al., 2017; Kim et al., 2015; Medford–Davis et al., 2017); Security (Kim et al., 2015; Medford–Davis et al., 2017); About secondary uses, unauthorized access, and errors regarding their EMRs (Hwang et al., 2012); Privacy and security of HIE, or security of EHRs (Dimitropoulos et al., 2011) **Facilitator**: Patient involvement in exchanging clinical records through a patient-based mechanism (Esmaeilzadeh, 2020a); Anonymization (Hunter et al., 2009).	**Benefits**: Fever infections, freely go outside, better information, protect me and others from COVID-19 (Habich-Sobiegalla & Kostka, 2022); Helping future patients and researchers (Nunes Vilaza et al., 2021); Improved care by allowing personalized treatment, by helping them understand their individual experience with Parkinson's, by helping their HC team understand their type of Parkinson's and by allowing monitoring of fluctuations and progression of the disease (Mursaleen et al., 2017); Improve the quality of both medical care and medical research (Kim et al., 2015); Improvement in their own and others' care (Soni et al., 2020); To help themselves and others with similar condition (Grando et al., 2017). **Concerns**: About privacy (Aggarwal et al., 2021; Krahe et al., 2019; Weng et al., 2019); About data misuse (Bouras et al., 2020; Krahe et al., 2019); About reidentification of anonymized health care data, and consent processes (Aggarwal et al., 2021); About private sector corporate interests, corruption, and profit making and expressed doubt about the Australian government's capacity to manage data sharing safely (Braunack-Mayer et al., 2021); About unethical projects, profit making without consent, and cyberattacks (Nunes Vilaza et al., 2021); Sharing data for commercial purposes regarding mental illnesses and with high de-anonymization risks (Calero Valdez & Ziefle, 2019); Risks with identifiable information, potential harm, unauthorized access to or sharing of information (Weng et al., 2019).
**Biobank data**	NA	**Benefits:** that biobank research will lead to improved health care (Critchley et al., 2012); Ninety-six percent of all respondents felt that SpecTRA's aim to develop a proteomic test for stroke was a worthwhile investment for health care, 98% said they were willing to provide a sample and/or information to facilitate the project's goals, and 96% to health research in general (Barnes et al., 2020). **Concerns:** About privacy (Ahram et al., 2022; Antommaria et al., 2018; Sanderson et al., 2017); About trust, and with data-sharing involving international researchers (Ahram et al., 2022); about data misuse (Sanderson et al., 2017) About potential discrimination, confidentiality breaches, and misuse of data for commercial or marketing purposes (Brall et al., 2021).
**Genetic and genomic data**	NR	**Benefits**: contributing to the greater good (Mählmann et al., 2017); discovery of a cure for untreatable diseases (84.3%) (Amorim et al., 2022) **Concerns**: about misuse of data, the fear of becoming a transparent citizen, and data safety (Mählmann et al., 2017); possible discrimination by health/life insurance companies and employers (Chavarria-Soley et al., 2021); lack of security and control over access to information, possibility of extracting information that exceeds the research objectives (Amorim et al., 2022); privacy (Parvinen et al., 2023)
**Miscellaneous data**	NR	**Benefits**: better treatments for others, data decrease in Swiss HC costs (Pletscher et al., 2022). **Concerns:** privacy (Jörling et al., 2023; Padrez et al., 2016; Pletscher et al., 2022); fear that sharing could affect employment (Padrez et al., 2016); potential identification despite anonymization, risk of higher health insurance premium and financial benefits for other people/companies (Pletscher et al., 2022)

Abbreviations: EHR, electronic health records; HIE, health information exchange; HC, healthcare; EMR, electronic medical record.

**Table 7:** Main socio-demographic and data-type specific factors that affect willingness to share.

Data type	Socio-demographic factors	Data-type specific factors
**Person-generated health data**	**Intention to share for primary use depends on the following variables:** **Lower intention** Older age (older than 55 years) (AORs 0.37–0.42, p < 0.01) (Bauer et al., 2017) Older age (older than 50), lower education degree (those having less than a bachelor's degree), lower income (Serrano et al., 2016); Older age (M = 3.84, SD = 0.12; F (2,107) = 7.439, p < 0.001) (Vervier et al., 2019). **Intention to share for secondary use depends on the following variables:** **Higher intention** Younger age (up to 34 years), (Pilgrim & Bohnet-Joschko, 2022); Male gender, and younger age (Cloos & Mohr, 2022).	**Intention to share for primary use depends on the following variables:** **Higher intention** Smartphone ownership and mobile health use (AORs 1.77–3.04, p < 0.01) (Bauer et al., 2017); Trust in information from their health professionals (Serrano et al., 2016); Higher health self-efficacy, higher level of trust in providers as a source of health information, higher level of physical activity, those with a higher frequency of wearable use, and who reported use of smartphones or tablets to help communicate with providers had greater odds of willingness to share data with providers (Rising et al., 2021).
**Personal health data and information**	**Intention to share for primary use depends on the following variables:** **Higher intention** Higher education level (β 5.0.18, p < 0.01) (Esmaeilzadeh, 2020b); Higher education (OR 2.17, 95% CI: 1.08–4.37) (Holderried et al., 2023); Age–with 1 year of increase in age, the log-odds of providing consent increases by 0.054 units (OR = 1.055; p < 0.0001), Female gender—for female patients, the odd of providing consent is 46% more than their male counterparts (OR = 1.460; p = 0.0003) (Yaraghi et al., 2015); Male respondents were more willing to share HD (OR = 2.2; 95% CI: 1.1–4.6) (Dhopeshwarkar et al., 2012); HC occupation (Itzhaki et al., 2023). **Intention to share for secondary use depends on the following variables:** **Higher intention** Younger age, female gender, HC occupation and region of residence (Europe and Middle East were more willing than North Americans and Asians) (Helou et al., 2021); Younger patients (≤49 years) were more uncomfortable than older patients (50 + years) with sharing data even within their own hospital (13% vs 2%, p < 0.05) (Tosoni et al., 2021); Younger age, higher education level (Karampela et al., 2019); Younger age - age group comparisons showed significant differences between the 75 and over age group and those in the 35–44 (χ2 (2) = 12.86, p = 0.002), 45–54 (χ2 (2) = 10.81, p = 0.004), 55–64 (χ2 (2) = 16.09, p < 0.001) and 65–74 (χ2 (2) = 19.75, p < 0.001) categories (Mursaleen et al., 2017); Increasing age, being retired and primary level of education were significantly associated with higher willingness to share: OR 1.39 (95% CI: 1.18–1.63), 2.00 (95% CI: 1.22–3.29) and 3.91 (95% CI: 1.95–7.85), respectively (Buckley et al., 2011); HC provider status–mothers with one HC provider (aOR 1.61, 95% CI: 0.95–2.73) increased their likelihood of willingness to share their EMR data with researchers by 2.7 times (Bouras et al., 2020); Higher education, White race (Kim et al., 2017).	**Intention to share for primary use depends on the following variables:** **Lower intention** Experiences of discrimination in the HC (OR 3.7; 95% CI: 2.6–5.2, p < 0.001), low trust in providers using health information responsibly (OR 2.3 95% CI: 1.4–3.6, p = 0.001) (Nong et al., 2022) Negative perceptions about the impact of EHRs (Kim et al., 2015) **Higher intention** Satisfaction with HC (OR 0.6 95% CI: 0.4–0.8, p = 0.001) (Nong et al., 2022); Trust in provider confidentiality (Iott et al., 2019); Patient–physician relationship and patient involvement (Abdelhamid et al., 2017); Trust in providers (Teixeira et al., 2011); Imposing safeguards to protect against unauthorized viewing, being able to see who has viewed their information, to stop electronic storage of their data, to stop all viewing, and to select which parts of their health information are shared (Dhopeshwarkar et al., 2012). **Intention to share for secondary use depends on the following variables:** Anonymity preferences (32.9% preferred anonymity, 28.2% of participants preferred pseudonymization) (Muller et al., 2022); Preferences for study-specific consent due to ethical concerns about potential research uses, and so they indicated a preference to be informed, educated, and given a choice (Tosoni et al., 2022); Perception about usefulness for public health research, data not being sensitive, and trust that their identity will remain anonymous after sharing it (Helou et al., 2021); Users, uses, data sensitivity, consent; government agencies and public institutions were the most trusted users of data (Lysaght et al., 2021); Respect of privacy, choices and needs for information regarding the use of participants' data (Courbier et al., 2019); Level of digitalization in their country (Karampela et al., 2019); Level of identification of data (Jung et al., 2020); Preference about being asked for permission health information use for any purpose other than medical treatment, and knowledge about data user (King et al., 2012); Being informed about their data being shared (Johansson et al., 2021); Being informed about who was using their data for what purposes, as well as about outcomes of the research (Kim et al., 2017); Type of use and obtaining consent (Grande et al., 2014); Data use, data user and data sensitivity (Grande et al., 2013); Sensitivity of information (Grande et al., 2015); Anonymity, research use, engagement with a trusted intermediary, transparency around PCHR access and use, and payment (Weitzman et al., 2010); Comfort level in sharing electronic health record data for personalized HC was highly correlated with sharing social media data (r = 0.78, p < 0.01) and sharing GPS location and text message data (r = 0.90, p < 0.01) (Garett & Young, 2022).
**Biobank data**	**Intention to share for secondary use depends on the following variables:** **Lower intention** Lower educational attainment—high school vs PhD, MD or similar (48%, OR 0.34, 95% CI: 0.20–0.53), religiosity (very religious vs not at all) (OR 0.77, 95% CI: 0.48–0.88) (Antommaria et al., 2018); **Higher intention** Younger people were more willing to share (Mezinska et al., 2020). Higher educational attainment (those with high school expressed lower intention to share vs those with PhD OR 0.47, 95% CI: 0.33–0.67, lower religiosity (“Very religious” participants were less willing to participate (63%) than “not at all religious” participants (OR 0.68, 95% CI: 0.54–0.85) (Sanderson et al., 2017). Self-identified white race (Black or African American participants expressed lower intention to share OR 0.59, 95% CI: 0.47–0.76) (Sanderson et al., 2017); Willingness to participate was higher in younger (18–24: aRP = 1.29, 95% CI: 1.12–1.49; 25–34: aRP = 1.16, 95% CI: 1.03–1.31) compared to older age groups (55–64; 65–74; 75–79), higher educated (those with compulsory education or less were less willing (aRP = 0.66, 95% CI: 0.55–0.80) whereas people with tertiary education were more willing (aRP = 1.24, 95% CI: 1.16–1.33) to participate), non-religious respondents (aRP = 1.17 95% CI: 1.04, 1.30) compared with very religious, and those with a background in the health sector (those who do not work aRP = 0.86, 95% CI: 0.80, 0.93 compared with those that work) (Brall et al., 2021).	**Intention to share for secondary use depends on the following variables:** No previous involvement in research and positive attitudes toward biobanks (Ahram et al., 2022); perceiving more research benefits, fewer concerns, and fewer information needs (Sanderson et al., 2017).
**Genomic data**	**Intention to share for secondary use depends on the following variables:** **Lower intention** Older age was significantly associated with a decreased willingness to share research data with non-profit organizations and any researcher who requests the information (OR = 0.96; 95% CI = 0.94, 0.99 and OR = 0.98; 95% CI = 0.97–1.00, respectively) (Goodman et al., 2017) **Intention to share for both primary and secondary use depends on the following variables:** **Higher intention** Younger age (people aged 30 and under), higher education level (those having a tertiary-level qualification) and race (those self-identified as White) (Middleton, Milne, Thorogood, et al., 2019); Younger age (up to 40 years), religiosity (non-religious respondents) (Romano et al., 2021)	**Intention to share for secondary use depends on the following variables:** Personal experience with genetics and genetic exceptionalist views (increased nearly six times the willingness to donate their anonymous DNA and medical information for research than other respondents) (Middleton, Milne, Howard, et al., 2019); Familiarity with the concepts of DNA, genetics, and genomics and trust in multiple actors were associated with willingness to donate DNA and medical information (Middleton et al., 2020); Having high levels of trust in all individuals/organization (OR 22.5, 95% CI: 15.5–32.5), having high levels of trust in medical professionals, moderate trust in university researchers and low trust in company researchers and own government) (OR 6.2, 95% CI: 5.2–7.4) (Milne et al., 2019). **Intention to share for both primary and secondary use depends on the following variables:** Being familiar with, or having a personal experience of, genetics/genomics (Middleton, Milne, Thorogood, et al., 2019)
**Miscellaneous data**	**Primary use:** **Higher intention was reported for the following variables:** Respondents' willingness to share “My dispensed medications” increased with age (Montelius et al., 2008) **Secondary use:** **Higher intention was reported for the following variables:** Older age (those aged ≥50 years), higher education, and vaccination status (being vaccinated against COVID-19 at least once, respondents who were ever vaccinated being 4.20 times more likely (95% CI: 3.21–5.48, p = 0.000) to be generally supportive of data-sharing than those unvaccinated) (Savic Kallesoe et al., 2023)	**Intention to share for primary use depends on the following variables:** Societal stigma strongly correlated with decreased non-psychiatric medication sharing, while self-stigma was strongly correlated with decreased psychiatric medications sharing (Yu et al., 2021); **Intention to share for secondary use depends on the following variables:** Higher willingness to share was associated with higher levels of satisfaction with the NHS, personal experience of mental illness, diagnosis with depression, obsessive-compulsive disorder, personality disorder or bipolar disorder (Kirkham et al., 2022); communication about prosocial benefit or social-life-enabling benefit of the app, higher perceived risk of the disease (Jörling et al., 2023)

Abbreviations: OR, odds ratio; aOR, adjusted odds ratio; CI, confidence interval; aRP, adjusted relative proportions; r, correlation coefficient; M, mean; SD, standard deviation; EHR, electronic health records; HC, healthcare; EMR, electronic medical record.

To assess the quality of the studies included in this review, the Newcastle–Ottawa Scale (NOS) was utilised. Two investigators independently evaluated the risk of bias, and in cases of disagreement, a third investigator was consulted for resolution. The NOS tool was employed to assess the quality of 116 non-randomised studies.

The NOS serves as an assessment tool specifically designed for evaluating the quality of non-randomised studies and aims to incorporate quality assessments into the interpretation of meta-analytic outcomes. It consists of seven categories for scoring and utilises a star system that is rolled up into three main themes: Selection (maximum of 4 stars across 4 sub-categories), Comparability (maximum of 2 stars across 1 sub-category), and Outcome (maximum of 3 stars across 2 sub-categories). The sub-categories are sample representativeness, sample size, comparability between respondents and non-respondents, ascertainment of exposure, comparability in terms of participant distribution and analyses, assessment of outcome, and statistical tests. For a comprehensive list of adapted questions from the NOS, please refer to the supplementary file provided (Supplementary Table S2).

All graphical presentations are generated by using Microsoft PowerPoint and Microsoft Excel software. To calculate the percentage of willingness to share HD across studies, the percentages across studies are pooled weighted by the number of respondents in each. The graphical presentation concerning willingness to share depending on the type of data and type of data recipient utilised bar charts, and to generate them, we extracted the total number of participants and the total number of those who were willing to share their HD from each study, and calculated the final percentage of those who were willing to share their data by using this formula:

(N willing to share (Study 1) + N willing to share (Study 2) + … + N willing to share (Study n))/(Total N (Study 1) + Total N (Study 2) + … + Total N (Study N)).

### Ethical approval

Ethical approval is not required for this particular systematic review as it relies on data obtained from previously published studies. Each of the studies incorporated in this review had already obtained ethical approval from their respective primary investigators before conducting the data collection.

### Role of the funding source

This study was not sponsored nor funded. All authors had full access to all the data in the study and accept responsibility for the decision to submit for publication.

## Results

### Search results

**Fig. 1:**
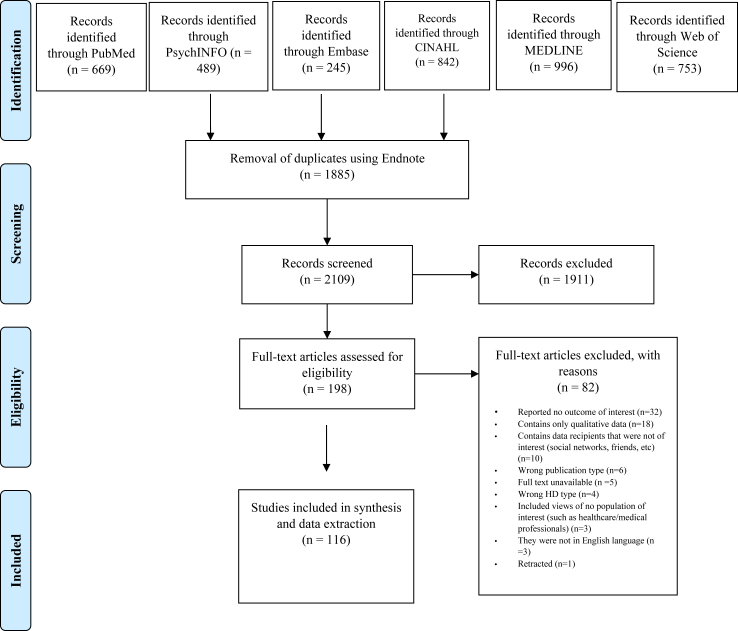
Preferred reporting items for systematic reviews and meta-analyses (PRISMA) study selection flow diagram.

The systematic literature search yielded a total of 3994 articles, obtained from the following databases: 669 articles from PubMed, 996 articles from MEDLINE, 489 article from PsycINFO, 245 articles from EMBASE, 842 articles from CINAHL, and 753 articles from Web of Science. After removing duplicate studies (n = 1396), a total of 2109 articles were screened based on title and abstract. A total of 1911 records were excluded after being deemed irrelevant for our research question, leaving 198 articles for full-text assessment. Of them, 67 articles were excluded due to following reasons: 32 reported no outcomes of interest, 18 reported only qualitative data, 6 were deemed to be of unsuitable publication type, 5 had no full-text available, 3 focused on views of populations not of interest, and 3 were published in a language other than English. Initially, 15 articles seemed to meet the inclusion criteria, however, after tabulating their information they were excluded as well: 10 reported HD sharing with recipients that were not of interest,^26^, ^27^, ^28^, ^29^, ^30^, ^31^, ^32^, ^33^, ^34^, ^35^ 4 reported wrong HD type or biospecimen only,^36^, ^37^, ^38^, ^39^ and 1 was retracted.^40^ This left 116 articles to be included in our qualitative summary, yielding a total of 228,501 participants (Fig. 1).

### Study characteristics

The main categorization of the findings from the studies identified in this systematic review was performed based on the type of data they were referring to, of which there are five subgroups. Within each subgroup, the results were then divided according to the type of use and were presented in the following order—first we presented findings related to sharing behaviours for primary uses, then for secondary uses, followed by for both primary and secondary uses. Survey-based studies were present the most (there were a total of 103 of them, and 8 either used vignettes or were defined as experimental), 11 were defined as mixed-method studies, 2 studies used discrete choice experiments. There were no RCTs identified, and only one survey study explored how willingness to share changed after undergraduate students were provided with educational material, which is the only study that explored how some kind of intervention influenced this variable.^41^ The subgroup of studies for reported behaviours related to the sharing of person-generated HD (PGHD) which represents only data that is generated by the patient (not providers) via home health equipment (such as those for blood glucose or blood pressure monitoring), via mobile device apps, wearable devices, or sensors (n = 17 and 10,771 participants, out of which 15 studies specifically referred to electronic data).^42^ Studies from the second subgroup reported sharing attitudes and behaviour of information that was described as PHID in general (n = 69 and 117,054 participants, out of which 46 studies referred specifically to electronic data). More details about the semantic issue of this subgroup of studies are provided in the corresponding Methods section. The third subgroup represents the studies that explored sharing intentions of biobank research data (n = 7 studies and 27,073 participants), the fourth focused on genomic data (n = 13 studies and 54,716 participants) and the fifth subgroup collected findings from studies that reported different types of data and were thus described as miscellaneous (n = 10 studies and 18,887 participants, out of which 7 specified referring to electronic type of data in specific). It should be noted that studies that investigated sharing genomic and biobank data did not make specifications on whether the data was electronic, however, as all these studies were published in the last decade, and the repositories are digital, we can presume that they are. The detailed characteristics of studies according to subgroups is given in Supplementary Table S2.

### Quality appraisal

The outcomes of the assessment of study quality with NOS are shown in Supplementary Table S3. The assessment results reveal the distribution of study quality based on the provided data. Among the studies evaluated, the majority displayed a moderate level of quality, specifically, there was 1 study rated with 3 stars, 6 studies with 4 stars, 7 studies with 5 stars, 18 studies with 6 stars, 20 studies with 7 stars, 38 studies with 8 stars, 17 studies with 9 stars and 9 studies with the highest score of 10 stars. These findings shed light on the varying levels of quality observed across the included studies and provide valuable insights into the overall rigor and robustness of the research conducted in this field.

### Studies reporting intention to share of person-generated health data

#### Attitudes, behaviour, willingness towards sharing person-generated health data

Studies focused on sharing person-generated health data (PGHD) for primary purposes reported relatively high willingness (Table 1), the highest being reported from a USA study on pregnant women (93%) that would share wearable device HD with their doctor^43^ and the lowest reported by a UK study (74%) that included 250 adults living with a range of long-term health conditions who would be willing to share health and lifestyle data with their healthcare professionals.^44^ There was a difference in willingness to share this type of data depending on the type of recipient (healthcare providers received more support,^45^ even compared with personal social circles)^44^, ^45^, ^46^ and the type of PGHD.^46^^,^^47^

Among the studies focusing on sharing PGHD (Table 1) for secondary purposes, the highest rate was reported by an Australian study (77%) on 101 Australian adults regarding sharing health and lifestyle data for research,^48^ and the lowest (48%) was for sharing stress data as reported by 29 participants from Sweden and Ireland (however, 72% of the participants expressed their intention to share fitness data).^49^ One German study also observed lower preferences for sharing with friends, than with scientists, general practitioners, or psychotherapists.^50^

**Fig. 2:**
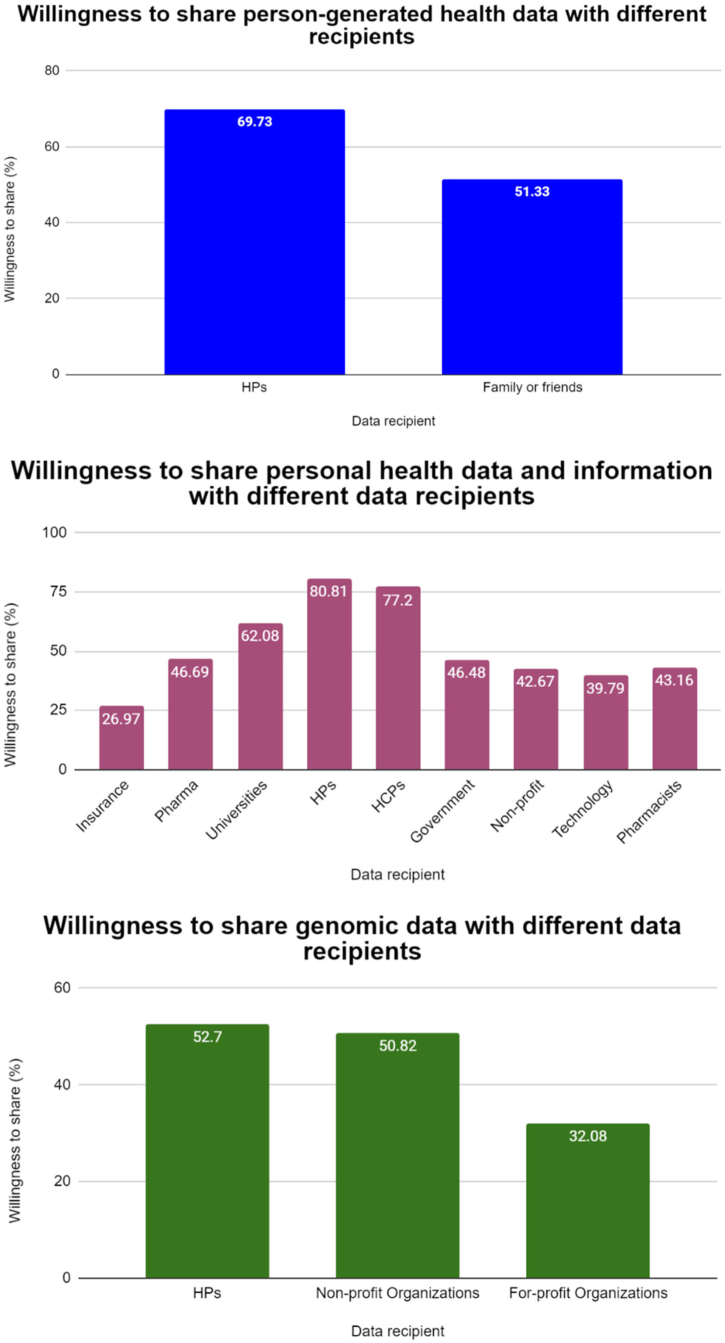
Quantitative summaries of willingness to share based on data type and recipient.

Studies focused on sharing for primary and secondary purposes (Table 1) revealed that doctors, universities, and public health institutions were viewed more favourably in terms of sharing PGHD compared with private or non-profit companies.^51^ The quantitative summaries of willingness to share depending on the type of data and on the type of data recipient are given in Fig. 2.

#### Benefits, concerns, and facilitators found to impact sharing person-generated health data

Concerns, benefits, and facilitators perceived by the study participants are presented in Table 6. Privacy was the most reported concern when sharing PGHD for primary uses,^46^^,^^52^^,^^53^ and transparency and privacy was considered significant in the case of sharing for secondary purposes.^43^^,^^48^ Study participants perceived they would gain benefits from sharing PGHD for primary purposes such as improvement in delivery and management of health care for them and others,^43^^,^^44^ while in the case of sharing for secondary purposes common good and better quality results were seen as beneficial.^54^

#### Sociodemographic and data-specific variables affecting sharing of person-generated health data

Among the included studies that reported sharing PGHD for primary and for secondary purposes, younger age was found to be a consistent significant factor that was associated with higher willingness to share.^47^^,^^49^^,^^53^^,^^55^^,^^56^ Other significant sociodemographic factors are listed in Table 7. For this group of data, reliance on mobile devices (including mobile health, smartphones, and wearables)^45^^,^^56^ was identified as a significant determinant of higher sharing intentions of study participants, among others.

### Studies reporting intention to share personal health data and information

#### Attitudes, behaviour, willingness towards sharing personal health data and information

Given that almost all of the other subgroups referred to electronic HD (studies reporting sharing of PGHD, PHID in general and biobanking data) and that the fifth category includes miscellaneous data (which disabled us from deriving conclusions also based on whether the data was electronic), this category had enough studies referring to both similar data with specifications about the use of the data (primary, secondary, or both) so we made a distinction in the presentation of results here.

Sharing PHID (Table 2) for primary purposes was reported to be highest in the case of USA patients with their treating physicians (95%),^57^ and the lowest by another study that included USA public regarding sharing their data with their healthcare providers (49%).^58^ One group of studies reported variability in sharing intentions depending on the data recipient, with findings indicating that healthcare providers that are in more direct relationships with the data donor/patient (such as doctors in charge, doctors they were referred to, healthcare providers working at the study sites), received higher support compared with other healthcare staff the participants have not encountered while receiving care. Studies also observed that sharing intentions differed based on the consent scenario,^18^ HIE architecture models,^59^ whether it was the case of emergency^19^^,^^60^ and whether healthcare providers implemented and utilised a direct exchange mechanism.^61^

Sharing PHID (Table 2) for secondary purposes was found to be highest in a study involving rare disease patients residing in the European Union (97%),^62^ while the lowest was found in a study that included USA healthy non-Hispanic white mothers (36%).^63^ The group of studies that distinguished sharing intentions depending on the data user (n = 14), consistently reported that institutions that conduct non-profit research (such as public hospitals, universities, other academic research institutions), receive higher support than organizations that are involved in for-profit and commercial research (pharmaceutical companies, insurance companies), or (health) government agencies. Willingness to share according to the type of data recipient is presented in Fig. 2. Three studies reported that data type determines one's willingness to share, and one of them observed that participants were in favour of sharing “traditional” health data as opposed to social media and device/app data.^64^ Other insights provided by these studies suggest that people categorized as belonging to the pandemic cohort were more comfortable with sharing their PHID compared with those belonging to the pre-pandemic cohort,^65^ and that privacy-preserving techniques can play an important role in someone's willingness to share.^66^^,^^67^

From the group of studies (Table 2) that reported how willingness to share for both primary and secondary purposes (n = 13), 12 reported that data recipients involved in their care (primary use of data) received higher support than those involved in secondary data uses (research institutions, pharmaceutical companies, and others). One study observed equal support for both healthcare and research (71%).^68^

#### Benefits, concerns, and facilitators found to impact sharing personal health data and information

Studies reported that study participants perceived sharing HD for primary purposes can bring benefitsthat are mainly directed to improvement of management and delivery of healthcare (including reduction of errors, better accuracy of their records and better communication with their providers). However, they also expressed concerns, and the most frequent ones are those related to privacy and safety of their data. Finally, this review identified that patient involvement in exchanging clinical records^61^ and anonymization^69^ could increase sharing intentions of HD (Table 6).

Among the studies focused on sharing intentions for secondary uses, the most frequently reported concern was again related to privacy (n = 6), followed by data misuse (n = 2). Study participants perceived that sharing their PHID for secondary purposes will bring benefits in the health and care system, and help both patients and researchers to better understand diseases and health conditions. These findings are presented in detail in Table 6.

#### Sociodemographic and data-specific variables affecting sharing of personal health data and information

Studies reporting sharing intentions of (electronic) PHID in general for primary purposes found associations with several sociodemographic variables (Table 7). For example, increased willingness was associated with achieving higher education,^67^^,^^70^ someone in a healthcare occupation,^71^ female gender,^72^ older age,^72^ and male gender.^59^ However, it should be noted that female gender was confirmed to be a significant predictor based on a study involving 20,076 participants, while the other study that found men to be more willing involved a much smaller sample size (n = 170). Among the studies that focused on sharing behaviours for secondary uses, younger age was found in four studies to be significantly associated with higher sharing intention (n = 4 and involved 112, 222, 8004 and 310 participants respectively),^73^, ^74^, ^75^, ^76^ while one study found the opposite (n = 1575).^77^ Higher education level was confirmed to be a significant predictor of increased willingness to share in two studies (n = 800 and n = 8004)^67^^,^^75^ while again Buckley et al., concluded the opposite (n = 1575).^77^

Significant variables that were identified to be relevant and specific for sharing PHID for primary purposes were related to satisfaction with and trust in healthcare, EHRs, healthcare providers and imposing adequate privacy safeguards. On the other hand, among the many enlisted data-specific variables regarding sharing PHID for secondary purposes, it can be observed that level of data identification, knowledge about data uses, users, and the level of data sensitivity and are very important factors that determine data sharing intentions (Table 7).

### Studies reporting intention to share biobank data

#### Attitudes, behaviour, willingness towards sharing biobank data

The willingness of sharing this type of data (Table 3) varied from as low as 10% in Arab countries,^78^ to very high of 96% among Canadian participants.^79^ Only one study reported parents’ willingness to enrol their youngest minor child in a hypothetical biobank and reported a rate of 55%.^80^

#### Benefits, concerns, and facilitators found to impact sharing biobank data

Studies reported the same concerns (Table 6) related to sharing biobank data as with the previously discussed data types: trust, privacy,^78^^,^^80^^,^^81^ and the misuse of data,^80^ while sharing data with international researchers concerned Arab people.^78^ However, study participants also perceived that sharing biobank data would lead to improvements in healthcare.^79^^,^^82^

#### Sociodemographic and data-specific variables affecting sharing of biobank data

We identified several sociodemographic predictors (Table 7) of higher willingness to participate in biobank research and donate data, of which higher educational attainment and lower religiosity was confirmed in two studies.^80^^,^^81^ Other significant data-specific predictors included: no previous involvement in research and positive attitudes toward biobanks,^78^ perceiving more research benefits, fewer concerns, and fewer information needs.^81^

### Studies reporting intention to share genomic data

#### Attitudes, behaviour, willingness towards sharing genomic data

The one study that reported genomic data sharing (Table 4) for primary purposes found that only around 55% of the USA public would consent to broad data sharing in a clinical setting.^83^ Willingness to share genomic data for secondary purposes (research) was highest among Norwegian newly discharged hospital patients (90%),^84^ and was the lowest among Swiss participants (55%).^85^ Four studies concluded that for-profit research received the lowest support. The only intervention study identified in this review observed a 24% increase in sharing genomic data for research among undergraduate students after educating them on the benefits.^41^ Studies that explored sharing genomic data for both primary and secondary purposes (part of the global online survey “Your DNA Your Say”) report relatively high willingness among the English-speaking publics from the UK, the USA, Canada, and Australia (67.77%)^86^ and Italy (64%).^87^ Willingness to share according to the type of data recipient is presented in Fig. 2.

#### Benefits, concerns, and facilitators found to impact sharing genomic data

The perceived benefits from sharing genomic data (Table 6) for secondary purposes were related to research advances and advancing treatments and cures.^85^^,^^88^ On the other hand, data safety, control, access, and the risk of being discriminated were perceived as important concerns.^41^^,^^85^^,^^88^^,^^89^ The studies that explored sharing for both primary and secondary purposes shared participants were afraid of their “DNA being copied and planted at the scene of a crime”, or their family and friends knowing something about them (data not shown).^86^

#### Sociodemographic and data-specific variables affecting genomic data sharing intention

Studies that explored (Table 7) sharing intentions for primary and secondary purposes observed that younger participants expressed a higher willingness to share their genomic data.^86^^,^^87^^,^^90^ In the case of sharing genomic data for both primary and secondary purposes, non-religiosity,^87^ White race and higher education level^86^ were significantly associated with higher sharing intentions.

For this type of data, familiarity with the concepts of DNA, genetics and genomics and trust in multiple actors,^91^^,^^92^ having personal experience with genetics and holding genetic exceptionalist views^86^ seem to play an important role in shaping public opinion towards sharing their genetic/genomic data.

### Studies reporting intention to share of data categorized as miscellaneous types of data

#### Attitudes, behaviour, willingness towards sharing health data categorized as miscellaneous

Studies that reported sharing attitudes of HD as miscellaneous (Table 5) for primary uses found that willingness to share medication records was higher when the recipient was a close relative or their physician (compared with the pharmacy and other health care staff),^93^ and that patients with multiple-sclerosis were more willing to share their non-psychiatric HD through their EMRs, compared with psychiatric HD.^94^

Studies that focused on sharing attitudes for secondary (research) purposes found the highest willingness to share clinical data among the adult Danish population (90%)^95^ and the lowest was observed among social media users, where 71% consented to share their social media data for the purposes of comparing it with their EMR.^96^

#### Benefits, concerns, and facilitators found to impact sharing miscellaneous types of health data

Studies that focused on sharing HD as miscellaneous for primary purposes did not report these outcomes. The perceived benefits (Table 6) related to sharing data for secondary purposes were seen in better treatment and decreased healthcare cost,^97^ while the most mentioned perceived concern was privacy.^96^, ^97^, ^98^

#### Sociodemographic and other variables affecting sharing intention of miscellaneous types of health data

Interestingly (Table 7), two studies concluded that older age was associated with a higher willingness to share HD—one focused on medication records for primary purposes^93^ and another focused on COVID-19 data for secondary purposes.^99^ Those with higher education levels and a positive COVID-19 vaccination status showed higher support for sharing COVID-19 data for secondary uses.^99^ Other data-specific factors included societal and self-stigma (in case of sharing non-psychiatric and psychiatric medication among patients with multiple sclerosis),^94^ higher levels of satisfaction with the UK's NHS, personal experience of mental illness (sharing mental health illness data),^100^ communication about prosocial benefit or social-life-enabling benefit of the app, and higher perceived risk of the disease (in case of sharing COVID-19 infection data to a tracing app).^98^

## Discussion

Sharing health data (HD) for primary purposes is a common experience for people to receive the best care. There are several categories of HD that can be part of the process of sharing information with health professionals, healthcare providers and health insurances including PGHD, PHID in general, genomic, and miscellaneous types of data such as medication records and psychiatric information, among the others identified throughout this systematic review. There is also a general need in many sectors of civil society for expanding the use of HD for secondary purposes such as medical research, health policies, public health purposes, national and international statistics, and personalized healthcare. However, the secondary use of HD points to the importance of public acceptance towards data sharing practices.^12^ This means that secondary use of HD should be aligned with the interests of the public (HD donors), and consequently it must be supported by the general population.

According to the evidence summarized in this review, people are generally very willing to share HD for primary purposes. This confirmation was derived from a sufficient number of studies that reported sharing intentions for PGHD and PHID, while only a single study was identified as relevant for this conclusion for genomic data but confirmed that people are moderately willing to share HD for primary purposes (55% were willing to do so). Biobank data did not apply as that data is oriented towards secondary uses only. Interestingly, several studies consistently found that people are more open to sharing their PGHD with healthcare providers compared with their inner social circle. Further, this group of studies point to the importance of different cofactors that inevitably shape an individual's willingness to share their data, such as the type of data recipient, the type of consent, type of HIE architecture, or whether it's a case of a health emergency. Although sharing for primary purposes is usually considered as an act that is expected to happen (regardless of any other influences) so the patient can receive the best care, the evidence says the opposite—sharing personal HD within the healthcare system is indeed quite complex.

The evidence concerning sharing data for secondary purposes is significantly different from the primary ones: it is clear the willingness to share is higher for primary than for secondary purposes and the difference mainly concerns specific data types. This is evident when inspecting the studies that assessed sharing intentions for both primary and secondary uses in the case of PHID–80% of studies concluded higher support in cases of sharing for primary uses. However, studies on sharing PGHD for secondary purposes report variable and often opposite findings among the general population, highlighting the need for more investigations. On the other hand, the number of studies on sharing PHID (electronic and non-electronic) that report higher rates of willingness to share for secondary purposes significantly outweigh those that report lower rates (75% vs 25%). It is interesting that both the highest and the lowest sharing rates for secondary purposes (among all studies) were observed in the case of biobanking data (98% and 10%, respectively). Sharing genomic data received relatively high support (65%) from two studies—one involving populations from several countries and one involving Italian general population (part of the project “Your DNA Your Say”).

Again, people's willingness to share their data for secondary uses is shaped by different factors, such as the type of consent model, the type of privacy-protection tools, the state of emergency (e.g., the COVID-19 pandemic), the type of data use, and what is most important the type of data user. Consistently, across all data types, non-profit organizations, and public institutions (universities, research institutes) received higher support than those that use data for making profit or are private (e.g., pharmaceutical companies). As expected, education and knowledge about data sharing is of great importance, however, there was only one intervention study involving Finnish undergraduate students, in which the rate of supportive students increased significantly after receiving education about sharing genomic data for secondary purposes. Thus more intervention studies are needed to confirm this suggestion.

**Fig. 3:**
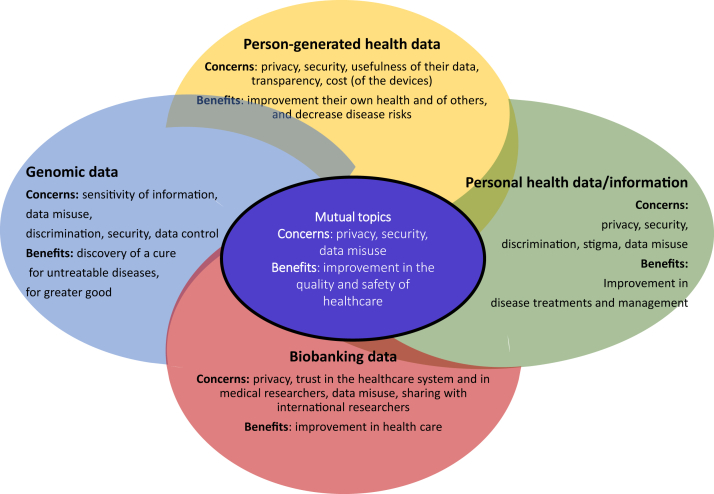
Comparative analysis of perceived concerns across all data types and misuse.

The opinions of study participants that reflect their concerns or perceived benefits regarding sharing their data, can either decrease or increase their intention to share. The comparison analyses of all studies revealed several mutual perceived concerns for all types of data (such as privacy and security) and data misuse (Fig. 3). On the other hand, perceived benefits that were mutual for all data types were related to improvement in healthcare (in specific aspects and in general). Evidence suggests that people need transparency and reliable privacy and security protection tools in order for them to be more open towards sharing their personal information.

This qualitative summary also identified what important sociodemographic factors determine willingness to share HD. In the case of sharing PGHD, younger age was consistently reported in several studies to significantly affect sharing this type of data for primary uses. Evidence from studies that reported sharing PHID for primary purposes found conflicting findings regarding the association between gender, education level and age. In the case of gender, the findings that suggest women are more open to sharing PHID was derived from a much bigger study sample compared with the other study suggesting the opposite (20,076 vs 170, respectively), indicating that female gender might be a more reliable predictor based on these two studies. Further, it can be suggested that both younger age and higher education level can be considered a more reliable predictor (than their counterparts), since they are derived from a higher number of studies (and consequently are based on a higher number of study participants). However, deriving such an accurate conclusion certainly requires a quantitative synthesis involving a meta-analysis as the most appropriate solution. Interestingly, younger age, higher educational attainment and being less religious were mutual sociodemographic predictors for sharing both biobank and genomic data. However, some of these insights were derived from individual studies, and thus these findings would benefit from additional studies that will confirm them.

Finally, the variables that were found to be specific for each data type should not be neglected. Such information can be used as information when addressing the needs of specific populations that were found to be more reluctant to share their data. For example, it was evident that people that are more reliant on mobile devices and smartphones are also more open to PGHD data sharing, compared with those who are not reliant. People who are not familiar with the concepts of genetics are also more hesitant to share their genomic data. As a matter of fact, only one intervention study was identified in this systematic review, and the evidence suggests that this research field would benefit from more RCTs (or similar intervention studies), that design specific interventions that can be used to address the concerns and barriers that people tend to have regarding sharing their data and potentially increase their willingness to share.

Sharing data is shaped by different ethical dimensions and can pose both positive and negative consequences. Findings from this qualitative synthesis confirm that the ethical aspect of data sharing is widely present in the opinion of data donors, and they perceive that sharing their data will bring benefits at the healthcare or the scientific level. Although people do acknowledge these benefits, they still lack understanding of different ethical aspects that relate to sharing practices. For example, it was seen from some studies that people would restrict the access to their data to specific parties (researchers that were in charge) or to specific uses (already defined in the protocol). However, many times the need for using their data may require different users and uses that are aligned with the final goal of reaching a better health-related outcome. All these details need to be presented in appropriate consent forms and institutional review board protocols^101^ and should be in accordance with the needs of both data donors and users. Thus, to ensure trust from data donors about sharing practices, they also need to be educated about what ethical data sharing involves.

On the other hand, there are ethical aspects concerning data donors that must be addressed in the data sharing process (which are mainly perceived as concerns—privacy, security, stigma, discrimination, among others). For example, based on the collected evidence, it is evident that privacy and security remain the most expressed concerns of data donors. Imposing adequate privacy-protecting tools (such as deidentification and anonymization) is found to alleviate these concerns (instead of asking participants to consent to using their identified data) which can bring positive consequences to data users (such as reduced research cost, increased recruitment rates, and decreased recruitment bias).^102^ On the other hand, it can bring considerable harm to the data donor in case their identity is revealed. It was evident throughout this review that transparency was an important concern and was a need that the participants expressed. Together with enforcing reliable protections, the public should also be informed about these activities and the corresponding consequences and benefits.

In line with the observations derived from this qualitative synthesis, a group of researchers from the University of Manchester found a high rate of people opting out of data sharing program after the government announcement of General Practice Data for Planning and Research (GPDPR) program in 2021, because people object to the patient-oriented constraints introduced by the GPDPR proposal (for example, patients are not allowed to decide whether their data should be shared, about the data recipient and the purpose of use^103^). There is a clear need for new regulatory approaches that will address the public's lack of trust in institutions, organizations, and the process of data sharing, that will enable higher transparency and find a more patient-centric solution that will provide them with more control without compromising the goals of HD use.

However, to fulfil its value, data sharing is suggested to follow the Findable, Accessible, Interoperable, Reusable (FAIR) principles.^104^ The problem with adhering to these principles occurs because data lacks standardization from the beginning. There is a clear need for exchange standards, domain-relevant content standards and accessible rich metadata, that will facilitate the interoperability in data sharing process, and consequently adherence to the proposed FAIR principles.^104^ Apart from those, there are challenges that need to be addressed, including technical, economic, legal, and political barriers. Datasets are constantly growing in their size and complexity, and are followed by incompleteness of data, lack of metadata and standards, lack of interoperability, among others. These issues are further amplified with the fact that different data types and their sources are often combined, in the necessity to overcome these challenges, which makes an even bigger problem because data sharing mechanisms are often data type specific.^105^ Robust standards and global collaborative efforts are necessary to address the barriers that hinder interoperability and the data sharing process.

On the lower level, data sharing strategists should consider potential barriers and challenges upfront, identify and then address them (before launching the final data sharing model). Some of them are enlisted in this review and can be utilised in defining the approach for addressing the needs of patients/public as stakeholders. This can be done by designing a practical minor intervention that will justify the value of the data-sharing program to the involved stakeholders (including professionals that act as data users and patients as data donors).^106^ Giving them knowledge and the (practical) implication about the benefits of their engagement in the data sharing process can ensure a better data sharing culture and motivation among different stakeholders.

Finally, we are going to point to strengths and limitations of this systematic review. Firstly, this review provides the first very detailed and thorough qualitative synthesis related to the issue of HD sharing attitudes that included all HD types identified in the published studies. It is the first to provide a comparison synthesis by making a distinction between primary and secondary uses and to make comparisons and conclusions for other variables and aspects that were found to significantly shape the will of people. It can be used as a map of available scientific evidence, which segments can be applied in designing both future (intervention) studies and policies that aim to tackle hesitancy and reluctance towards sharing HD where there is an identified need for this. Given its wide inclusion criteria, the limitations of the synthetized evidence are expected. Firstly, the dominance of the studies that investigated sharing of (electronic) PHID is evident, and their number significantly outnumbers studies that investigated other types of data; therefore, future studies should take this into consideration. Further, the evidence synthetized in this form does not allow us to derive straightforward conclusions regarding the differences in sharing intentions based on the type of study population, gender, age, education level. It provides an overview of this evidence, however, an analysis focusing on specific aspects with the attempts to quantify the potential associations is necessary for making straightforward conclusions. Another limitation of this data is also the inhomogeneity of their presentation (some reported odd ratios, other test statistics depending on the difference in the percentages, etc), and this was another hindrance in making accurate and reliable conclusions based on their summaries.

In conclusion, an individual's willingness to share health information can be viewed as a result of weighing both positive and negative factors that are related to the process of sharing personal health information. Positive or negative preferences and attitudes are significantly driven by different factors^107^ such as types of health data or information, privacy concerns, information on security level, results of health information use, altruism, illness histories and others. Tools such as gaining consent, data anonymization, and establishing regulations for gaining access to data could facilitate and encourage data sharing. All these factors shape what we call the culture of health data sharing. It's interesting under this perspective that people tend to change their attitude towards data sharing and data use depending on whether they consider the secondary research ‘acceptable’ (e.g., health service) and ‘unacceptable’ (e.g., commercial) research.^15^, ^16^, ^17^ Possibly, this change can be driven better by an improved culture and education allowing a safe and promising development of scientific, commercial, and public health purposes. This should also inspire policy makers in requiring guidelines and data regulations—which are now missing—that promote a well-informed, evidence-based and transparent public communication on health data uses, which is fundamental to create awareness and trust in health data sharing for beneficial purposes, further improving our healthcare system and outcomes.

## Contributors

F.C. conceived the research hypothesis. F.C. and A.P. designed the study. A.P. and Y.A.A. performed the article screening. A.P., V.P. and L.D.M, performed the data extraction and the quality assessment. All authors had access to data and verified it (F.C., A.P., Y.A.A., V.P., LDM, W.R.). F.C. and W.R. has shaped the manuscript with input from the entire team (written contributions of single paragraphs).

## Data sharing statement

The review protocol is publicly available on PROSPERO. The data was collected from publicly available databases and is available with this publication.

## Declaration of interests

The authors have no competing interest to declare.

## Appendix A Supplementary data

Supplementary Files Revised
