# Supplementary material for: Health data sharing attitudes towards primary and secondary use of data: a systematic review
Source: eClinicalMedicine. 2024 Mar 18;71:102551. doi: 10.1016/j.eclinm.2024.102551 (PMC10963197; doi:10.1016/j.eclinm.2024.102551)
Supplement: Supplementary Files Revised [file mmc1.pdf]

Supplementary Table 1. Literature search keywords

**Journal Databases Keyword Strategy**<br>Search Keywords
("health data" OR "patient data" OR "medical data" OR "health information" OR “person generated health data” OR "electronic health records" OR “mobile apps” OR “apps” OR "wearable technology" OR "wearable device" OR "big data")
**AND**<br>("sharing" OR "data sharing" OR “health data sharing” OR "health information exchange")
**AND**<br>("attitudes" OR "beliefs" OR "perceptions" OR "willingness")

Supplementary Table 2. Results of Newcastle Ottawa Scale (NOS) assessment for quality appraisal of non-randomiszed studies.

Study	Total Score	Selection				Comparability	Outcome	
		Representativeness of the sample	Sample size	Non-respondents	Ascertainments of exposure	Based on design and analysis	Assessment of Outcome	Statistical Test
Abdelhamid et al., 2021	7	*	*		*	**	*	*
Abdelhamid et al., 2017	8	*	*		*	**	**	*
Aggarwal et al., 2021	6	*	*		*	*	*	*
Ahram et al., 2022	5	*	*			*	*	*
Alaqra and Kane 2020	6	*	*		*	*		*
Amorim et al., 2022	8	*	*	*	*	**	*	*
Ancker et al., 2012	7	*	*	*	*	*	*	*
Antommaria et al., 2018	8	*	*	*	**	*	*	*
Barnes et al., 2020	6	*	*	*	*		*	*
Bauer et al., 2017	8	*	*	*	**	*	*	*
Bakken et al., 2022	4	*			*	*		*
Bosanac and Stevanovic 2022	4		*		*	*		*
Bouras et al., 2020	8	*	*		**	**	*	*
Braunack-Mayer et al., 2021	6	*	*		*	*	*	*
Brown et al., 2022	8	*	*		*	**	**	*

Buckley et al., 2011	8	*	*	*	*	**	*	*
Chavarria-Soley et al., 2021	7	*	*		*	**	*	*
Chen et al., 2016	6	*	*		*	*	*	*
Cloos et al., 2022	8	*	*	*	*	**	*	*
Courbier et al., 2019	6	*	*		*	*	*	*
Critchley et al., 2012	9	*	*	*	*	**	**	*
Dimitropoulos et al., 2011	8	*	*	*	*	**	*	*
Dhopeshwarkar et al., 2012	7	*	*	*	*	*	*	*
Drake et al., 2022	6	*	*		*	*	*	*
Eikemo et al., 2022	7	*	*	*	*	*	*	*
Esmaeilzadeh 2020	6	*	*		*	*	*	*
Esmaeilzadeh 2020	8	*			**	**	**	*
Fylan and Fylan 2021	6	*	*		*	*	*	*
Garret and Young 2022	6		*	*		*	*	*
Goodman et al., 2017	9	*	*		**	**	**	*
Grande et al., 2015	9	*	*	*	**	**	*	*
Grande et al., 2013	9	*	*	*	**	**	*	*

Grande et al., 2014	10	*	*	*	**	**	**	*
Grando et al., 2017	7	*	*		*	*	*	*
Habich-Sobiegalla and Kostka 2021	8	*	*		*	**	**	*
Hartmann et al., 2019	6	*	*		*	*	*	*
Heidel et al., 2021	7	*	*		**	*	*	*
Helou et al., 2021	4				*	*	*	*
Holderried et al, 2023	10	*	*	*	**	**	**	*
Holm et al., 2021	10	*	*	*	**	**	**	*
Hunter et al., 2009	7	*	*		*	**	*	*
Hwang et al., 2012	6		*	*	*	*	*	*
Itzhaki et al., 2023	7	*		*	*	**	*	*
Kim et al., 2017	7	*	*	*	*	*	*	*
Luo et al., 2020	6	*			*	*	**	*
Lott et al., 2019	10	*	*	*	**	**	**	*
Jones et al., 2016	7	*	*	*	*	*	*	*
Jorling et al., 2022	9	*	*	*	*	**	**	*
Jung et al., 2020	7	*	*	*	*	*	*	*

Karampela et al., 2019	7	*	*	*	*	*	*	*
Kaufman et al., 2016	8	*	*	*	*	**	*	*
Kim et al., 2015	7	*	*	*	*	*	*	*
Kim et al., 2017	9	*	*	*	*	**	**	*
Kim et al., 2019	8	*	*	*	*	**	*	*
Kimura et al., 2014	8	*	*		*	**	**	*
Kimura et al., 2022	4	*		*	*	*		
King et al., 2012	8	*	*	*	*	**	*	*
Kirkham et al., 2022	5		*		*	*	*	*
Kongeter et al., 2022	5	*	*	*	*		*	
Krahe et al., 2019	6		*		*	*	**	*
Ly et al., 2022	9	*	*	*	*	**	**	*
Lysaght et al., 2021	8	*	*	*	*	*	**	*
Mählmann et al., 2017	3	*	*		*			
Mahmoud et al., 2019	6	*	*		*	*	*	*
Maus et al., 2021	5	*	*		*	*		*

Medford-Davis et al., 2016	10	*	*	*	**	**	**	*
Middleton et al. 2020	7	*			*	**	**	*
Middleton et al. 2019a	9	*	*	*	*	**	**	*
Middleton et al. 2019b	8	*	*		*	**	**	*
Milne et al., 2022	9	*	*	*	*	**	**	*
Montelius et al., 2008	9	*	*	*	*	**	**	*
Muller et al., 2022	8	*	*	*	*	*	**	*
Mursaleen et al., 2017	8	*	*	*	*	*	**	*
Nong et al., 2022	9	*	*	*	*	**	**	*
Nunes et al. 2021	10	*	*	*	**	**	**	*
Parvinen et al., 2023	8	*	*	*	*	**	*	*
Patel et al., 2011	9	*	*	*	*	**	**	*
Patel et al. 2012	9	*	*	*	*	**	**	*
Patil et al. 2016	8	*	*	*	*	**	*	*
Pedersen et al. 2015	8	*	*	*	*	**	*	*
Perera et al. 2011	9	*	*	*	*	**	**	*
Pilgrim & Bohnet-Joschko 2022	7	*	*	*	*	*	*	*

Riggs et al., 2019	8	*	*	*	*	**	*	*
Riordan et al. 2015	10	*	*	*	**	**	**	*
Rising et al., 2021	8	*	*	*	*	**	*	*
Romano et al., 2021	8	*	*	*	*	*	**	*
Mezinska et al. 2020	8	*	*	*	*	*	**	*
Runkle et al. 2019	8	*	*		*	**	**	*
Sanderson et al., 2017	9	*	*	*	**	**	*	*
Savic Kallesoe et al., 2023	9	*	*	*	*	**	**	*
Serrano et al. 2016	7	*	*		*	*	**	*
Soni et al. 2020	8	*	*	*	*	**	*	*
Teixeira et al. 2011	4				*	*	*	*
Tosoni et al. 2021	8	*	*	*	*	**	*	*
Tosoni et al. 2022	10	*	*	*	**	**	**	*
Trachtenbarg et al. 2017	8	*	*	*	*	**	*	*
Trinidad et al. 2020	6	*	*		*	*	*	*
Ziefle and Valdez, 2018	6	*	*		*	*	*	*
Velarde et al. 2021	5	*	*		*	*	*	

Vervier et al. 2019	8	*	*	*	*	*	**	*
Johansson et al. 2021	10	*	*	*	**	**	**	*
Vidgen et al 2020	8	*		*	**	*	**	*
Wang et al., 2019	5	*	*		*	*	*	
Weitzman et al., 2012	8	*	*	*	*	**	*	*
Weng et al., 2019	8	*	*	*	*	**	*	*
Whiddett et al. 2016	5	*			*	*	*	*
Whiddett et al., 2006	8	*	*	*	*	**	*	*
Yaraghi et al., 2015	4				*	*	*	*
Yu et al., 2021	8	*	*	*	*	*	**	*
Ziefle and Valdez 2018	7	*	*		*	*	**	*
Corman et al., 2022	7	*	*	*	*	*	*	*
Padrez et al., 2016	8	*	*	*	*	**	*	*
Pletscher et al., 2022	7	*	*	*	*	*	**	*
Soni et al. 2019	9	*	*	*	*	**	**	*
Weitzman et al., 2010	6	*	*		*	*	*	*
Woldaregay et al. 2020	8	*	*	*	*	**	*	*

A full list of NOS adapted questions

- **Representativeness of the sample**. *

The sample is clearly or somewhat representative of the general population or the population of interest.

- **Sample Size**. – Justified and satisfactory. *

The appropriate sample size for a study assessed using the NOS is not predetermined, **as the scale does not require a specific sample size**. The adequacy of sample size is evaluated based on the precision of the estimates, the confidence intervals, the effect sizes, and the statistical significance of the results.

- **Non-respondents. ***

When comparability between respondents and non-respondents' characteristics is established, and the response rate is satisfactory.

- **Ascertainments of exposure –**

When measurement tool is validated**.** **

When measurement tool is not validated but the tool is available or described. *

- **Comparability**

The subjects in different outcome groups are comparable, based on the study design or analysis. Confounding factors are controlled.
a) The study controls for the most important factor (e.g age, sex etc). *
b) The study control for any additional factor. *

- **Assessment of Outcome**

Independent blind assessment. **
Record linkage. **
Self-report. *

- **Statistical test**

The statistical test used to analysze the data is clearly described and appropriate, and the measurement of the association is presented, including confidence intervals and the probability level (p value). *

Supplementary Table 3. Study characteristics of the included studies, per subgroup.

Name of the subgroup	Number of studies per type of use	Data types reviewed in the specific subgroup	Geographical locations of the included studies
Studies reporting behavior related to sharing of person-generated health data (n=17)	Primary n=9, secondary n=6 and primary and secondary n=2	Heath data tracked via mobile devices (n=4), wearable devices and sensors (n=4), general self-tracked health data (n=3), self-tracked health and lifestyle data (n=3), patient-generated data (n=1), fitness tracked data (n=1), wearable device and stress data (n=1), data from apps for the self-monitoring and self-management of depression (n=1)	The USA (n=7), Germany (n=5), UK (n=1), Australia (n=1), Sweden (n=1), Sweden and Ireland (n=1), and international (Switzerland, Norway, USA, UK, Australia, Canada, France, and Germany) (n=1)
Studies reporting behavior related to sharing of personal health data/information (n=69)<br><br>Note: 6 studies were entered as separate records reporting results on HD sharing intentions separately for	Primary n=26, secondary n=37, primary and secondary n=12	Primary: PHID in general (n=2), electronic PHID (n=3), electronic PHID for HIE (n=9), for e-health (n=1), from EHRs (n=7), from EMRs (n=3), from PHRs (n=1);<br><br>Secondary: PHID in general (n=17),	The USA (n=32), Canada (n=4), Australia (n=4), China (n=2), UK (n=3), Germany (n=3), New Zealand (n=3), South Korea (n=2), Israel (n=1), Switzerland (n=1), England (n=1), Ireland (n=1), Denmark (n=1), Singapore (n=1), Croatia (n=1), Norway (n=1), and 10 were international.

primary and secondary purposes		PHID from medical records (n=1), PHID and biospecimen (n=1), electronic PHID (n=3), from EHRs (n=9), from EMRs (n=5), from PCHRs (n=1); Primary and secondary: PHID in general (n=3), electronic PHID (n=2), from EHRs (n=3), from EMRs (n=1), from PHRs (n=1), from PCHRs (n=1), via e-health (n=1)	
Studies reporting behavior related to sharing of biobank research data (n=7~~6~~)	All were for secondary purposes (research)	Biobank data	The USA (n=2), Switzerland (n=1), Latvia (n=1), Canada (n=1), Australia (n=1), 4 Arab countries (Jordan, Egypt, Morocco and Sudan, n=1)
Studies reporting behavior related to sharing of genomic data (n=13)	Primary n=1, secondary n=9, primary and secondary n=3	Genomic data	UK, USA, Canada and Australia (part of the global online survey entitled "Your DNA Your Say", n=3), the USA (n=2), Switzerland (n=1), Norway (n=1), Costa Rica (n=1), Portugal (n=1), Finland(n=1), Australia (n=1), the USA (n=2), Italy ("Your DNA Your Say", n=1), international (22 countries, "Your DNA Your Say", n=1)
Studies reporting behavior related to sharing of HD defined as miscellaneous (n=10~~2~~)	Primary n=2, secondary n=9, primary and secondary n=1	Clinical data (n=2), COVID-19 infection data (n=2), medication records, psychiatric health information, clinical trial data, mental health data, de-identified medical images, social media data and emergency medical record data, emergency medical record data	Canada (n=3), USA (n=2~~3~~), ~~Canada (n=3),~~ Germany (n=1~~2~~), Switzerland (n=1), UK (n=1), Denmark (n=1), Sweden (n=1).
